# Modeling nature‐based restoration potential across aquatic–terrestrial boundaries

**DOI:** 10.1111/cobi.70046

**Published:** 2025-04-28

**Authors:** B. Wegscheider, N. K. Rideout, W. A. Monk, M. A. Gray, R. Steeves, D. J. Baird

**Affiliations:** ^1^ Aquatic Ecology and Evolution, Institute of Ecology and Evolution University of Bern Bern Switzerland; ^2^ Department of Fish Ecology and Evolution, Centre for Ecology, Evolution and Biogeochemistry Swiss Federal Institute of Aquatic Science and Technology (EAWAG) Kastanienbaum Switzerland; ^3^ Environment and Climate Change Canada, Canadian Rivers Institute, Department of Biology University of New Brunswick Fredericton New Brunswick Canada; ^4^ Environment and Climate Change Canada, Canadian Rivers Institute, Faculty of Forestry and Environmental Management University of New Brunswick Fredericton New Brunswick Canada; ^5^ Canadian Rivers Institute, Faculty of Forestry and Environmental Management University of New Brunswick Fredericton New Brunswick Canada; ^6^ Fisheries and Oceans Canada, Gulf Fisheries Centre Moncton New Brunswick Canada

**Keywords:** biodiversity, conservation prioritization, cross‐ecosystem function, rewilding, watershed, biodiversidad, cuenca hidrológica, función interecosistémica, priorización de la conservación, resilvestración

## Abstract

Today, few watersheds remain untouched by global change processes arising from climate warming, impoundments, channelization, water extraction, pollution, and urbanization. The need for restoration has resulted in a myriad of interventions, generally performed at small scales, which have limited measurable impact in restoring biodiversity and ecosystem functions. We propose bringing nature‐based restoration (also referred to as rewilding) principles to rivers and their watersheds to allow freshwater ecosystems to heal themselves and present a case study example for the Wolastoq, a transboundary watershed on North America's east coast. We aimed to identify key areas for the provision of the ecosystem function secondary productivity in the watershed and explored how the existing network of protected lands contributes to its conservation. We first developed species distribution models for 94 aquatic insects and 5 aerial insectivores and then considered human footprint and existing protected areas when employing spatial prioritization to meet 2 area‐based targets (17% and 30% [i.e., Aichi Biodiversity Target 11 and Canada's 30×30, respectively]) for conservation or restoration of freshwater secondary production. Current conservation protection in the watershed was predicted to be insufficient to protect either ecosystem function providers or receivers of secondary production. By considering integrated conservation strategies, restoration and conservation actions can be better allocated throughout habitat patches to ensure sustained provision of ecosystem functions across the watershed. Nature‐based restoration and conservation can help inform Canada's area‐based targets, providing a framework for incorporating ecosystem functions into conservation planning and offering practical insights for policy and restoration efforts aimed at safeguarding biodiversity.

## Graphical abstract

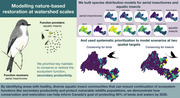

## Abstract (es)

Modelos del potencial de restauración basado en la naturaleza a través de los límites acuático‐terrestres

**Resumen:**

Hoy en día, son pocas las cuencas hidrográficas que permanecen al margen de los procesos de cambio global derivados del calentamiento climático, los embalses, la canalización, la extracción de agua, la contaminación y la urbanización. La necesidad de restauración ha dado lugar a un sinfín de intervenciones, generalmente realizadas a pequeña escala, que tienen un impacto mesurable limitado en la restauración de la biodiversidad y las funciones de los ecosistemas. Proponemos aplicar los principios de la restauración basada en la naturaleza (también denominada resilvestración) a los ríos y sus cuencas para que los ecosistemas de agua dulce puedan curarse a sí mismos, y presentamos un ejemplo de estudio de caso para el Wolastoq, una cuenca transfronteriza de la costa este de Norteamérica. Nuestro objetivo era identificar las áreas clave para la provisión de la función ecosistémica de productividad secundaria en la cuenca y explorar cómo la red existente de tierras protegidas contribuye a su conservación. Primero desarrollamos modelos de distribución de especies para 94 insectos acuáticos y 5 insectívoros aéreos y después consideramos la huella humana y las áreas protegidas existentes al realizar la priorización espacial para cumplir dos objetivos basados en el área (17% y 30% [es decir, el objetivo de biodiversidad 11 de Aichi y el 30 x 30 de Canadá, respectivamente]) para la conservación o restauración de la producción secundaria de agua dulce. Se predijo que la protección actual de la conservación en la cuenca era insuficiente para proteger a los proveedores de funciones ecosistémicas o a los receptores de producción secundaria. Si se tienen en cuenta las estrategias de conservación integradas, las acciones de restauración y conservación pueden distribuirse mejor entre las zonas de hábitat para garantizar el suministro sostenido de funciones ecosistémicas en toda la cuenca. La restauración y la conservación basadas en la naturaleza pueden contribuir a los objetivos canadienses basados en la superficie, proporcionando un marco para incorporar las funciones de los ecosistemas a la planificación de la conservación y ofreciendo ideas prácticas para las políticas y los esfuerzos de restauración destinados a salvaguardar la biodiversidad.

## INTRODUCTION

Biodiversity is declining at an alarming rate, corresponding to a disruption of ecosystem functions and erosion of ecosystem resilience (Alves & Rosa, 2007; Felipe‐Lucia et al., 2020). Despite these trends, habitat restoration initiatives often narrowly concentrate on individual species rather than addressing the broader spectrum of ecological processes and functions that diverse species communities provide (Miller & Hobbs, 2007). This limited focus ignores the interconnectedness of species that provide and respond to ecosystem functions in functioning communities, therefore limiting the potential success of restoration efforts. Urgent action is needed to shift restoration strategies to more holistic approaches that prioritize the restoration of ecological processes and functions to safeguard biodiversity. Although some suggest that habitat types be separated for purposes of reporting on conservation status (e.g., Juffe‐Bignoli et al., 2016), we favor an integrated approach that works at larger spatial scales. This reflects the realities of terrestrial–freshwater linkages for watersheds in terms of species’ habitat use and critical ecosystem functioning across boundaries, including nutrient and biomass flux (Ballinger & Lake, 2006).

In Canada, 2 area‐based conservation targets have been proposed to address biodiversity loss. In 2010, Canada adopted the Aichi Biodiversity Target 11 for the protection of 17% of terrestrial and marine ecosystems, set as a national target for implementation by 2020 (Hagerman & Pelai, 2016). More recently, in 2022, Canada—alongside 190 other countries—committed to Kunming–Montreal Global Biodiversity Framework (GBF) goals to conserve 30% of its land and waters by 2030 (30×30) (United Nations Convention on Biological Diversity, 2022). Despite these goals, by 2024, <14% of Canada's terrestrial and freshwater ecosystems had been protected (ECCC, 2024b), and to date, only general guidelines on plans for 30×30 have been provided (ECCC, 2024a). Additionally, the largest protected areas are in Northern regions that lack many of the anthropogenic stressors related to development facing watersheds in other ecozones (ECCC, 2024b). Although these goals are largely conservation focused via protection targets, restoration can and should play a part in these area‐based targets, ensuring continued provisioning of healthy source populations and ecosystem functioning at wider spatial scales.

The integration of conservation and restoration for contribution to area‐based targets can be aided by systematic planning. Systematic conservation planning tools aim to prioritize areas of high biodiversity (Leal et al., 2020), focal taxa (Tozer et al., 2020), or ecosystem services (Dai et al., 2023; Vaz et al., 2021) while minimizing costs (such as area amount or cost of purchasing land) (Margules & Pressey, 2000). Conservation planning has been biased toward terrestrial ecosystems (Jung et al., 2024), neglecting crucial freshwater habitats and aquatic–terrestrial ecosystem processes that bridge these habitats (Linke et al., 2019). These linkages underpin the functioning of both aquatic and terrestrial ecosystems, influencing nutrient cycling, water quality, and species interactions (Hladyz et al., 2011). Recently, researchers have explored the use of conservation planning tools in concert with restoration targets to prioritize the multifunctionality of ecosystem services in riverine floodplains (Tschikof et al., 2024) and to identify priority areas for cross‐taxa interactions (Nogueira et al., 2023). Yet, there remains a notable deficit of restoration projects that prioritize the restoration of ecosystem functions (but see Thierry & Rogers, 2020).

Conservation of land for the protection of biodiversity requires going beyond the identification of biodiversity hotspots for 2 reasons. First, these do not currently add up to enough land to meet current policy targets (see above) or may not be sufficient for endemic or range‐restricted species (e.g., Shrestha et al., 2019). Second, the importance of large‐scale intactness at the regional or watershed scale underlines the importance of knowing the restoration potential of unconserved areas in terms of their current state and proximity to existing conservation areas. Conserved areas provide critical resources to support nature‐based restoration and build out to the surrounding region. Indeed, many protected areas are created with the goals of reducing human pressures and thereby healing and restoring vulnerable ecosystems (e.g., land held in stewardship by environmental nongovernmental organizations). Moreover, in changing ecosystems and climates, integration of conservation and restoration can help move beyond the categorization of goals and approaches and toward action, including through the process of rewilding (Wiens & Hobbs, 2015).

Rewilding has been proposed as a powerful restorative force at large, even watershed scales (Rideout, Wegscheider, et al., 2021). Although trophic rewilding actions target the restoration of megafauna (Saveedra et al., 2023), passive rewilding actions focus on helping humans reduce environmental pressures so that nature can heal at its own pace (Holmes et al., 2020). Although the necessity and pragmatism of rewilding actions will differ geographically, according to regional and local contexts, the first step will require an assessment of the rewilding potential of a system (Ceaușu et al., 2015; Perino et al., 2019). Rideout, Wegscheider, et al. (2021) created a conceptual rewilding framework for rivers, based on the restoration and protection of essential ecosystem functions at a watershed scale. This explicitly differed from widespread traditional restoration that focused on physical riverine structures or focal species at small scales, rather than ecosystem functions. The holistic approach of rewilding as a restoration action can help functioning areas in watersheds support restoration areas in damaged or otherwise less functional parts of a system. We use the term *nature‐based restoration* throughout this article to keep the spirit of passive rewilding while recognizing that there are concerns about the ethics of continuing to use the term *rewilding* in North America given the significant colonial history of associating the wild with the explicit and active exclusion of Indigenous Peoples from their traditional lands and waters (Schulte to Bühne et al., 2022) (see also Hayward et al. [2019], Derham et al. [2019], and Anderson et al. [2019] for debates on the nuances and usefulness of the term *rewilding*).

Because nature‐based restoration is broad in scale and scope, the identification of areas suitable for either conservation or restoration can support wider watershed functioning and continued provisioning of ecosystem functions at larger spatial scales. For example, the nature‐based restoration framework created by Rideout, Wegscheider, et al. (2021) included 12 ecosystem functions, consisting of functional, biotic, and structural components. Here, we applied this framework to the Wolastoq watershed, on Canada's east coast, and focused on the ecosystem function secondary production, through the energy transfer of aquatic insects (function providers) to terrestrial ecosystems, particularly aerial insectivores (function receivers). When aquatic insects emerge with the metamorphosis from larvae to adults, they bring a flux of nutritionally important fatty acids to riparian and terrestrial ecosystems (Twining et al., 2016). Riparian predators, particularly aerial insectivores (birds that capture insects on the wing, including flycatchers, swifts, swallows, and nightjars), are especially reliant on these insects during breeding periods (Twining et al., 2018). This avian group is of conservation concern due to its drastic population declines across North America, which are particularly evident across eastern Canada (Spiller & Dettmers, 2019). Despite the importance of freshwater secondary production as a resource base for aquatic and terrestrial consumers, productivity is not currently included in freshwater monitoring programs in Canada. Because they are a key group contributing to freshwater secondary production, we focused on emerging aquatic insects—known to be important food resources for freshwater and terrestrial at‐risk species, such as aerial insectivores (Shipley et al., 2022; Stenroth et al., 2015).

We used spatial prioritization to evaluate 8 scenarios to meet 2 area‐based targets (17% and 30%) to conserve or restore important areas for the ecosystem function secondary production in the Wolastoq watershed in Atlantic Canada. Our scenarios prioritized key habitats for aquatic insects as ecosystem function providers and 5 representative species of aerial insectivores as ecosystem function receivers. We also examined the contribution of existing protected lands in the watershed to selected areas. Our objectives were to identify key areas for aquatic insect richness that can provide source populations and services for areas undergoing nature‐based restoration; examine how well aerial insectivores are represented in scenarios prioritizing habitat for ecosystem function providers (aquatic insects) and vice versa; and examine how well existing protected lands in the watershed contribute to the conservation of ecosystem function provision.

## METHODS

### Study system

**FIGURE 1:**
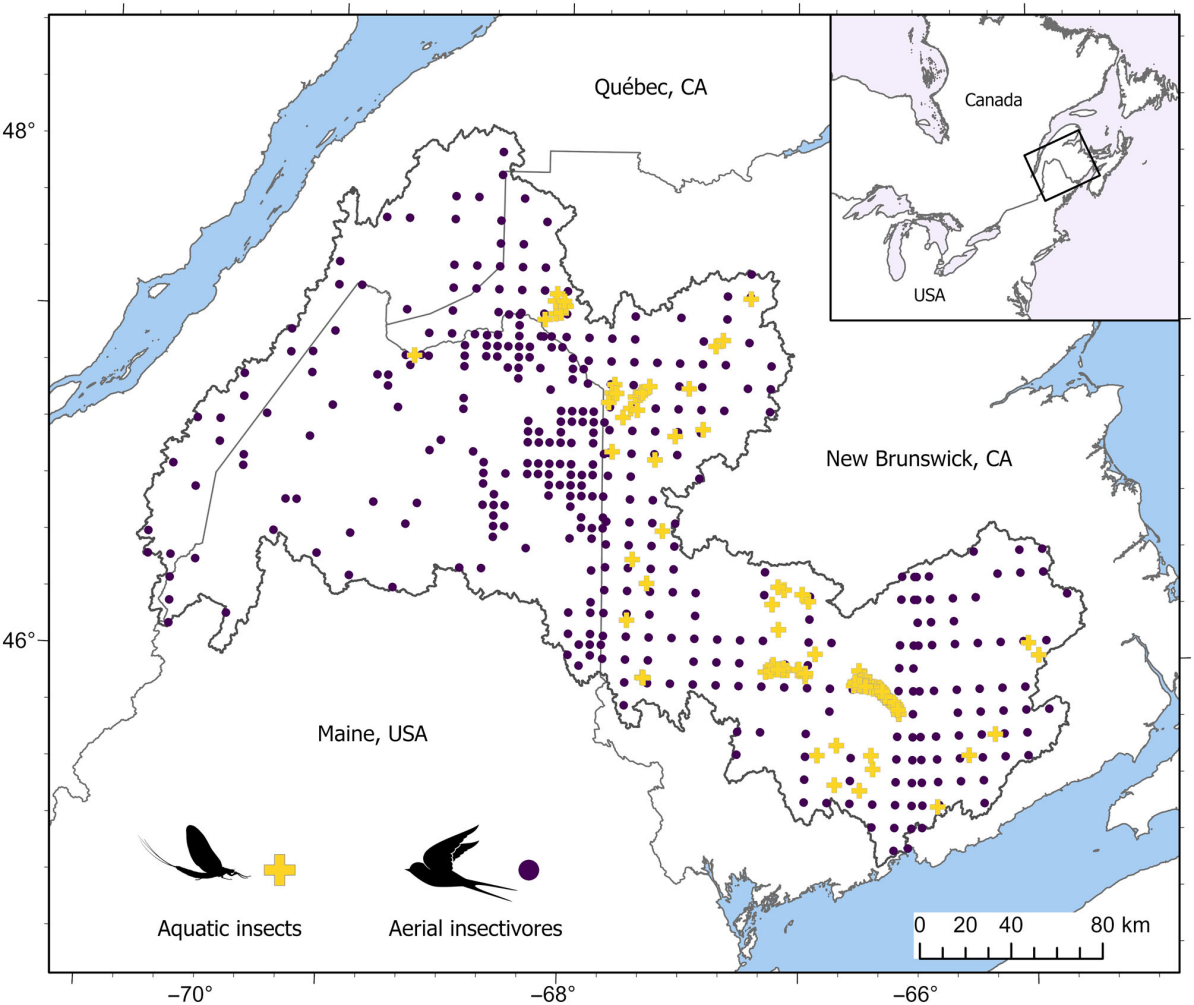
Location of the sites for the aquatic insect eDNA community samples and records of the 5 aerial insectivores used as inputs for species distribution models.

We chose the Wolastoq (Figure 1) (also known as the Saint John River [*fleuve Saint‐Jean*]) as a case study watershed to apply the nature‐based restoration framework proposed by Rideout, Wegscheider, et al. (2021) because of its conservation value; encroaching forestry, mining, and energy developments; and availability of open‐source data. A transboundary river bordering the United States and Canada, the Wolastoq drains a catchment of 55,110 km^2^ as it flows 673 km from its headwaters in northern Maine and Quebec through New Brunswick before emptying into the Bay of Fundy (Cunjak & Newbury, 2005). Despite possessing one of the last semi‐intact floodplains on the eastern coast of North America, it currently experiences a range of human pressures and stressors, including multiple large hydroelectric dams, urbanization, forestry, agriculture, and climate change (Kidd et al., 2011; WWF‐Canada, 2017). Habitats in the watershed were rated as inadequately protected or not protected at all by the recent World Wildlife Fund (WWF) Wildlife Protection Assessment (WWF‐Canada, 2019); however, the Canadian federal government has recently designated the Wolastoq a priority place for conservation and restoration due to its ecological significance (Government of Canada, 2022). Numerous local watershed groups have performed hundreds of small‐scale restorations across the catchment, including bank and riparian vegetation restoration and removal of small dams and barriers (e.g., WWF‐Canada, 2020).

### Biodiversity and ecosystem functioning

Insect data were drawn from 2 separate collections for the New Brunswick portion of the watershed. One is focused on riverine and the other on floodplain wetland macroinvertebrates. Both data sets were built using overlapping eDNA markers that target insects. The river benthic macroinvertebrate samples were collected for the Government of Canada's Genomics Research and Development Initiative's (GRDI) Ecobiomics Project (Edge et al., 2020) following CABIN (Canadian Aquatic Biomonitoring Program) wadeable streams protocols (Environment and Climate Change Canada, 2012). With this protocol, 3‐min kick samples were taken with a 400‐µm net. The samples were rinsed in the field to remove excess sediment and stored in 95% ethanol. In the laboratory, all vegetation was rinsed and removed with a 25‐µm sieve, and ethanol was replaced to minimize dilution. Samples were stored at −80°C and subsequently transported while frozen to the Department of Fisheries and Oceans lab in Moncton, New Brunswick, for DNA extraction and amplification of the cytochrome c oxidase subunit 1 (COI) barcoding region. The FWH2 (Elbrecht & Leese, 2017; Vamos et al., 2017), BR5, and a modified version of the F230 amplicon were used in the metabarcoding (Gibson et al., 2015), and library preparation involved 2 separate steps of amplification before amplicons were size‐selected, pooled, and sent to the National Research Centre's Sequencing facility in Saskatoon for 2×250 bp Illumina MiSeq sequencing. For the floodplain wetland samples, we used data from Rideout et al. (2022a), who followed the CABIN wetland macroinvertebrate protocol (Environment and Climate Change Canada, 2019). For further details on processing and DNA metabarcoding analysis, see Rideout et al. (2022b).

Details of the target DNA sequences used for components of the combined data set used in this study and detailed molecular protocols of the Ecobiomics project are in Appendix S1. For all sequence data generated, taxonomic classifications were assigned using the Ribosomal Database Project classifier 2.12 (Porter & Hajibabaei, 2018). These data were filtered for >99% confidence for correct assignments at the genus level. Data generated from DNA metabarcoding were treated as presence–absence information (Elbrecht & Leese, 2015). We further filtered the invertebrate data set exclusively for insect taxa because these taxa emerge following metamorphosis and transition from aquatic to terrestrial ecosystems; they are considered function providers of secondary productivity in this study.

We focused on 5 focal species of aerial insectivores known to forage for emerging aquatic insects in the watershed: tree swallow (*Tachycineta bicolor*), barn swallow (*Hirundo rustica*), cliff swallow (*Petrochelidon pyrrhonota*), eastern phoebe (*Sayornis phoeb*e), and eastern kingbird (*Tyrannus tyrannus*). We acquired data from the Quebec Breeding Bird Atlas (Québec Breeding Bird Atlas, 2014), the Maritimes Breeding Bird Atlas (New Brunswick data [Bird Studies Canada et al., 2012]), and the Maine Breeding Bird Atlas (Maine Department of Inland Fisheries & Wildlife, 2022 [accessed through eBird, 2023]). Data in the Quebec and Maritimes Breeding Bird Atlases are collected by highly trained volunteers (community scientists confident in bird identification and calls), who count species seen or heard in a 5‐min period across a minimum of 15 roadside sites randomly distributed in 10‐km grid squares. The Maine Breeding Bird Atlas also relies on trained community science volunteers, but their surveys are distributed across 31 regions in the state. The data are entered using eBird, where time and distance traveled are accounted for as effort in the final data set. For all atlases, volunteers are encouraged to focus on adding new species with each subsequent survey in their square to best capture species richness and remove the bias of common species. We treated all atlas data sets as presence‐only observations and pooled all data at a 10‐km grid square level to reduce bias from human population density. We considered aerial insectivores function receivers of secondary productivity.

### Environmental data

We derived a drainage network of the Wolastoq based on a Shuttle Radar Topographic Mission digital elevation model (Farr et al., 2007) and modeled overland flow accumulation, resulting in 20,067 river segments and corresponding subcatchments with an average area of 2.71 km^2^. Next, we created a suite of landscape variables to be used in our models as covariates that are expected to be highly influential on the distribution of aquatic and riparian species along river networks. We included a range of hydroclimatic, land‐use, and topographic conditions by compiling the following variables for each river segment and reach‐contributing area: average slope, average annual temperature, average temperature of the warmest quarter, total annual precipitation, total precipitation of the warmest quarter, proportion of deciduous tree cover, proportion of needleleaf tree cover, proportion of mixed forest cover, proportion of grassland cover, proportion of wetland cover, proportion of cropland cover, and proportion of urban cover. Climate variables were derived from the CHELSA database (Karger et al., 2017, 2021), and land‐cover classes were compiled from the North America Land Change Monitoring System (NALMS; CCRS et al., 2023). We harmonized environmental data to a common 120 × 120‐m raster grid with the New Brunswick Stereographic projection (NAD83(CSRS)/New Brunswick Stereographic). Watershed delineation and covariate processing were performed in R 4.2.2 (R Core Team, 2022) with openSTARS package 1.2.3 (Kattwinkel et al., 2020).

### Species distribution modeling

We developed species distribution models (SDMs) based on species occurrence, climate, and biophysical data sets for aquatic insects and aerial insectivores. To avoid collinearity, selection of environmental covariates was reduced using the variance inflation factor in the usdm R package 1.1‐18 (Naimi et al., 2014). For aquatic insects, we excluded genera that were present at fewer than 10 sites, resulting in 120 taxa. The SDMs for these taxa were considered presence or absence. For aerial insectivores, we focused on the 5 species outlined above, and the models were considered presence only due to the nature of sampling.

We fitted random forest models for each taxa using environmental data as covariates and taxa presence (and absence, where applicable) as response variables. Random forests perform well at prediction tasks across multiple data types and perform equally to model ensembles for species distribution modeling (Waldock et al., 2022). A major benefit of random forests is automatically recovering nonlinearities and variable interactions (Valavi et al., 2021). To compute predictive power, we performed 5‐fold cross‐validation, in which sampling units were assigned randomly to 5 folds, and predictions for each fold were based on models fitted to data on the remaining 4 folds (Appendix S2). We examined the explanatory and predictive performance of the random forest models through 4 evaluation metrics, focusing on the species‐specific area under the receiver operating characteristic curve (AUC), true skill statistic (TSS), Cohen's kappa, and accuracy (Appendix S2). The TSS ranges from −1 to +1 (values >0 indicate models performing better than at random) and was used to retain genera with sufficient model performance for further analyses. We retained models of genera with a TSS score of at least 0.3 (total = 94 insect genera) (Appendix S2). Successful models for 94 genera were then projected with environmental data across the watershed, yielding probabilistic maps of a taxa's presence that range from 0 (lowest probability) to 1 (highest probability) at a 120‐m grid resolution. To approximate aquatic insect genus richness in the watershed, we transformed probability of occurrence models to binary outputs using the TSS evaluation metric as a cutoff value. These binary outputs were combined to stacked SDMs representing the distribution of aquatic insect genus richness in the watershed. Finally, we analyzed how richness changes across different environmental gradients in the watershed. All analyses of both taxonomic groups, aquatic insects and aerial insectivores, were performed using the biomod2 R package 4.2‐3 (Thuiller et al., 2024).

### Systematic prioritization analyses

**FIGURE 2:**
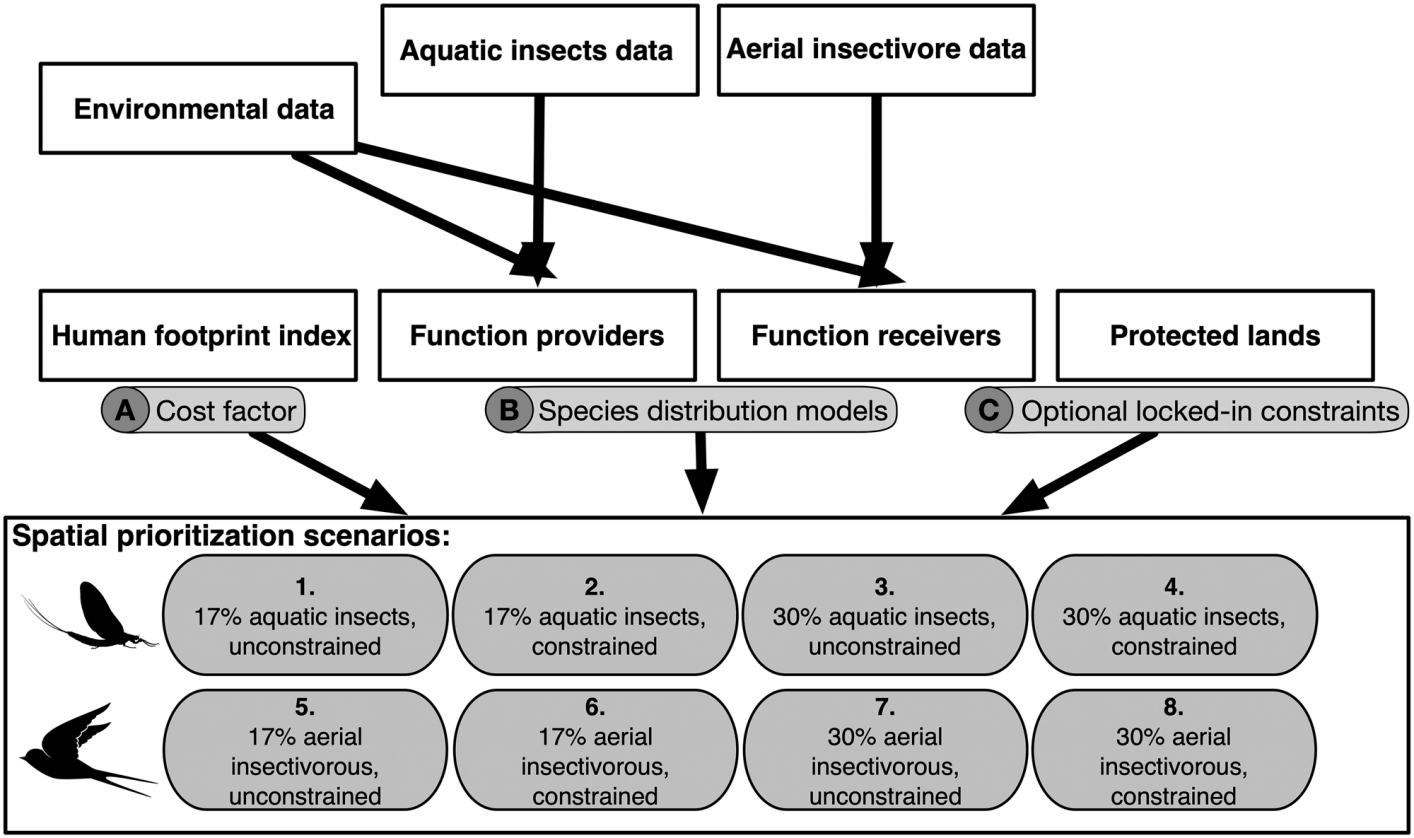
Workflow describing inputs for spatial prioritizations and 8 scenarios for conservation and restoration of secondary productivity.

In the conservation prioritization analysis, we used Canadian Hydrographic Units data (Nature Conservancy of Canada, 2019) as watershed conservation planning units for the Wolastoq (*n* = 412). We used the taxa distributions of 94 aquatic insect taxa and 5 aerial insectivores in the Wolastoq catchment as our conservation features. Our model setting considered cost minimization (i.e., minimum set problem), representation targets of 17% and 30% for conservation features, and scenarios where protected watershed units were included as locked‐in constraints (Figure 2). For the cost parameter, we used the watershed‐specific component of the Watershed Health Assessment for the Northern Appalachian–Acadian Region of Canada, a human footprint index based on 17 spatial data sets characterizing human disturbances known to both directly and indirectly affect freshwater biodiversity (e.g., non‐native fish species, nutrient and contaminant point source discharges, land‐use categories, unpaved road densities) (Millar et al., 2019). The human footprint index was developed using the first principal component axis of the standardized data extracted for each level of the Canadian Hydrographic Units subcatchments representing a gradient from forestry activity through to a more agricultural and urban signature (Millar et al., 2019).

Throughout the exercise, we considered a watershed unit as protected when more than 30% of its area included protected lands (Millar et al., 2019), considering provincial parks, provincial protected areas, national wildlife areas, and state conserved lands. We ran 8 scenarios (Figure 2), prioritizing habitat for either function providers or function receivers, with either 17% or 30% area targets, and considering where existing protected lands were included (constrained) or not (unconstrained). We compared the overlap of prioritized habitats with existing protected lands and examined trade‐offs in area representation between the different scenarios. The exact solution to each problem was obtained by setting the optimality gap, representing the acceptable deviance from the optimal objective at 10% (Beyer et al., 2016). The solution was binary, wherein each planning unit was either selected or not in the resulting spatial design. We tested whether the conversion of probability to binary values resulted in the selection of different priority areas and derived summary statistics from a confusion matrix for a quantitative comparison (Appendix S3). To evaluate the relative importance of individual planning units in achieving representation targets of conservation features, we computed irreplaceability scores (*sensu* Ferrier et al., 2000). We conducted the prioritization analysis with the prioritizr package 8.0.3 (Hanson et al., 2023) in R 4.2.2 and the optimization solver Gurobi (Gurobi Optimization). All figures and maps were created using the ggplot2 3.4.4 (Wickham, 2016) and tmap 3.3.4 (Tennekes, 2018) packages. R scripts for analyses and figure generation are found in Wegscheider (2024).

## RESULTS

### Key areas of aquatic insect richness and provisioning of secondary production as an ecosystem function

**FIGURE 3:**
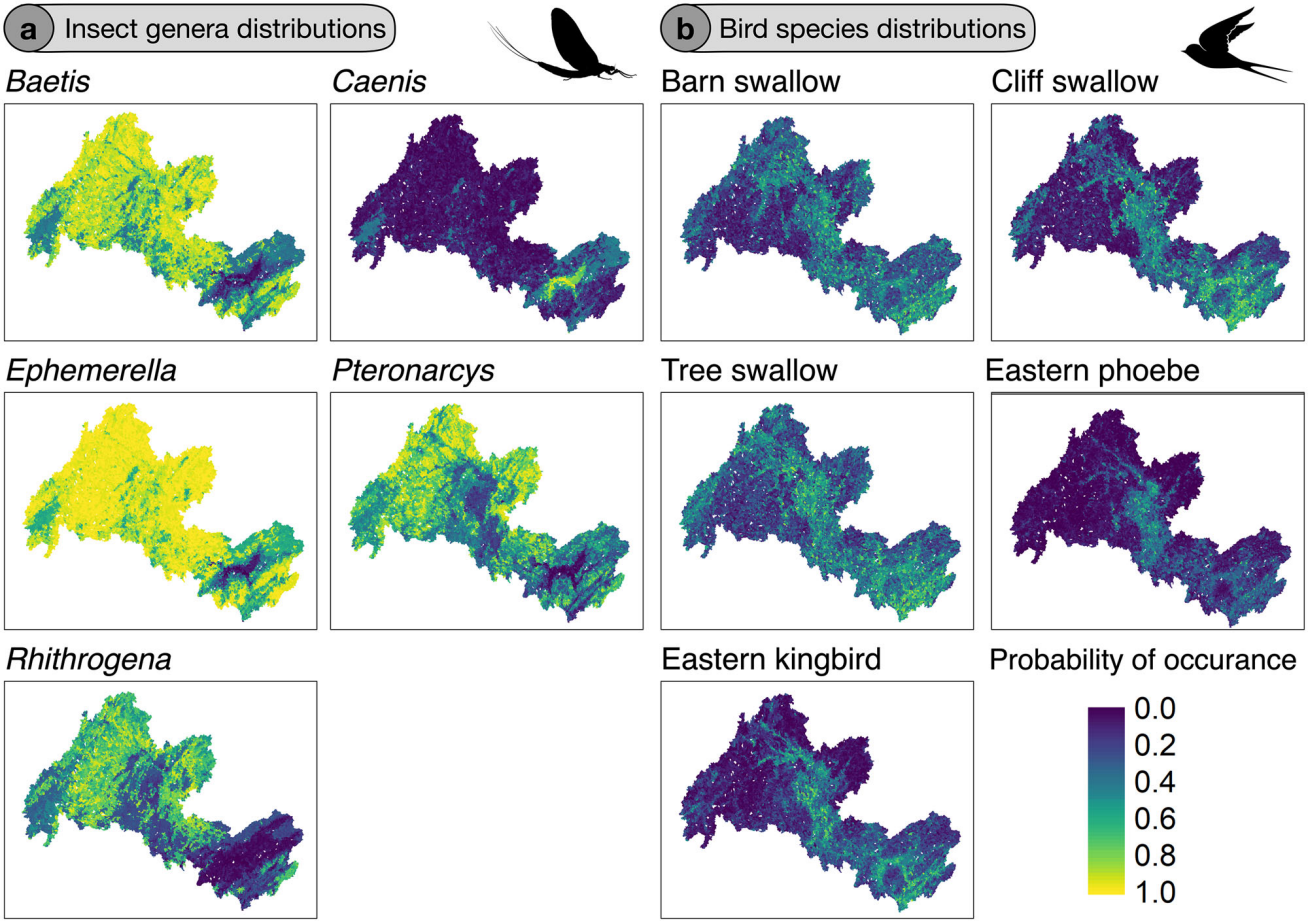
Estimates of species distributions of (a) 5 example genera of aquatic insects and (b) 5 aerial insectivores in the Wolastoq watershed, Canada (color gradient from blue to yellow indicates an increase in habitat).

**FIGURE 4:**
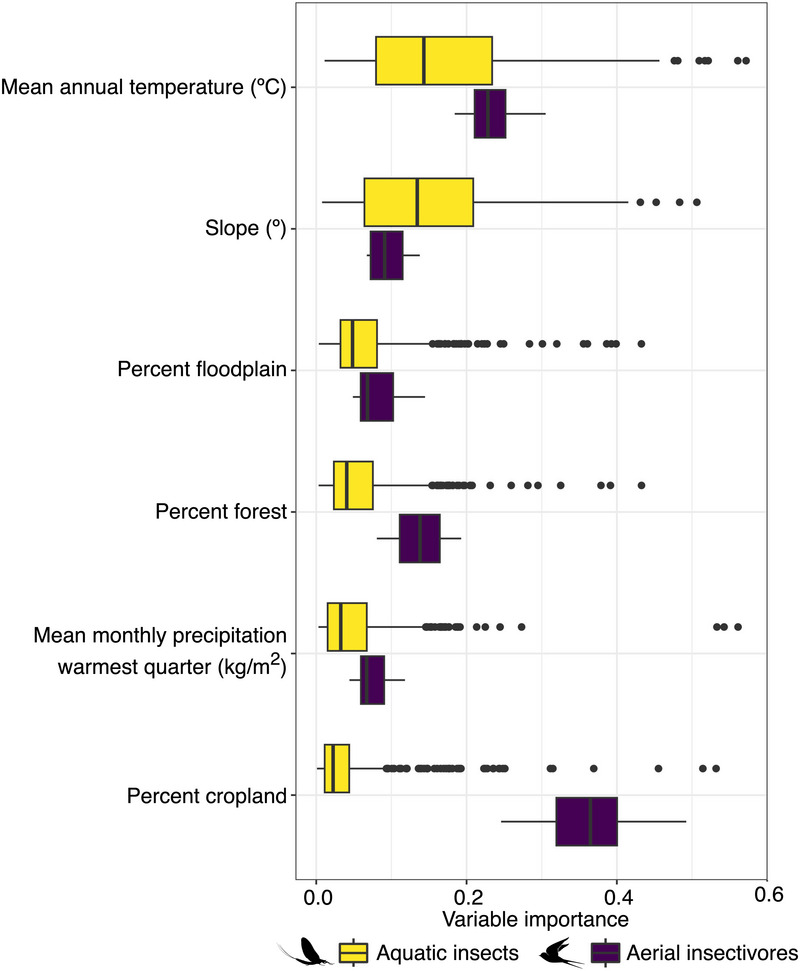
Variable importance scores for aquatic insects and aerial insectivores used for building species distribution models.

The highest habitat suitability for aquatic insects was generally found in headwater regions of the watershed, whereas the lower portion of the Wolastoq was more suitable for taxa associated with depositional (sedimentary) areas, such as *Caenis* (see Figure 3 as a reference with example genera). Mean annual temperature and slope were identified as the 2 covariates with the highest variable importance score in SDMs (Figure 4). Both temperature and slope showed a strong gradient from small, steep, and cold streams in headwater regions to warmer main stem and larger tributaries in the lower portion of the watershed. Similarly, there was a strong gradient in genus richness of aquatic insects, ranging from up to 70 genera in the headwater region to fewer than 10 in the downstream, lower Wolastoq (Appendix S4). These strong patterns were associated with an increase in richness in colder environments, steeper channels, and a higher proportion of forested areas, whereas an increased proportion of floodplains and cropland showed a negative relationship to genus richness (Appendix S4).

Compared with aquatic insects, aerial insectivores showed contrasting spatial patterns in the distribution of suitable habitats. For all 5 species, there tended to be more suitable habitat along the main stem of the Wolastoq and its major tributaries, including the large floodplain complex in the lower portion of the watershed (Figure 3). In contrast to aquatic insects, the percent coverage of cropland in reach‐contributing areas had a strong, generally positive effect on explaining the distribution of aerial insectivores, whereas slope and precipitation were consistently weaker (Figure 4). Although all 5 species tended to occur with a higher probability in locations with a higher percentage of floodplain habitat (Figure 3), the percentage of floodplain was a relatively weak contributor to overall model performance (Figure 4).

### Representation of ecosystem function providers and receivers in different scenarios

In Table 1, we summarized how well habitats of the function providers and receivers were represented in the existing conservation network and each prioritized solution, namely examining the representation of all 94 aquatic insects and each aerial insectivore. In all scenarios and solutions, the 17% and 30% area‐based conservation targets were met for all conservation features (Table 1). For the 17% area‐based target, representation of aquatic insects averaged at 19.4% and 18.3% with a maximum of 24% and 20% for the constrained and unconstrained scenarios, respectively. For the 30% area‐based target, average representation was 31.8% and 31.6% with maximum coverage of 35.8% and 36.1% for the constrained and unconstrained scenarios, respectively. Among the 5 aerial insectivores, cliff swallows had the highest representation at the 17% and 30% area‐based target, with 19.38% and 34.33% of their distribution represented in the unconstrained solution, respectively (Table 1). The other aerial insectivores were all slightly above the 17% and 30% target in both constrained and unconstrained solutions. Because species varied in the extent of their distribution, there were considerable differences in selected areas, ranging from 0.08 and 0.16 km^2^ for eastern phoebe to 0.20 and 0.35 km^2^ for tree swallows, to meet the 17% and 30%, respectively.

**TABLE 1:** Amount of species distribution range represented by existing protected areas only and in solutions of each prioritized scenario with the 17% and 30% area‐based targets.

		17% target		30% target	
Taxa	Coverage by existing protected areas (%)	Constrained (%)	Unconstrained (%)	Constrained (%)	Unconstrained (%)
94 aquatic insect genera (mean)	10.6	21.9	18.9	34.0	32.9
Barn swallow	4.9	18.2	17.7	31.2	31.2
Cliff swallow	3.8	19.0	19.7	32.7	33.4
Tree swallow	5.2	18.0	17.1	30.4	30.4
Eastern phoebe	3.2	17.5	19.6	31.4	32.6
Eastern kingbird	4.7	18.6	18.3	31.6	31.1

*Note*: Constrained scenarios have conserved land watersheds locked into the solutions, and unconstrained scenarios have no constraints.

**FIGURE 5:**
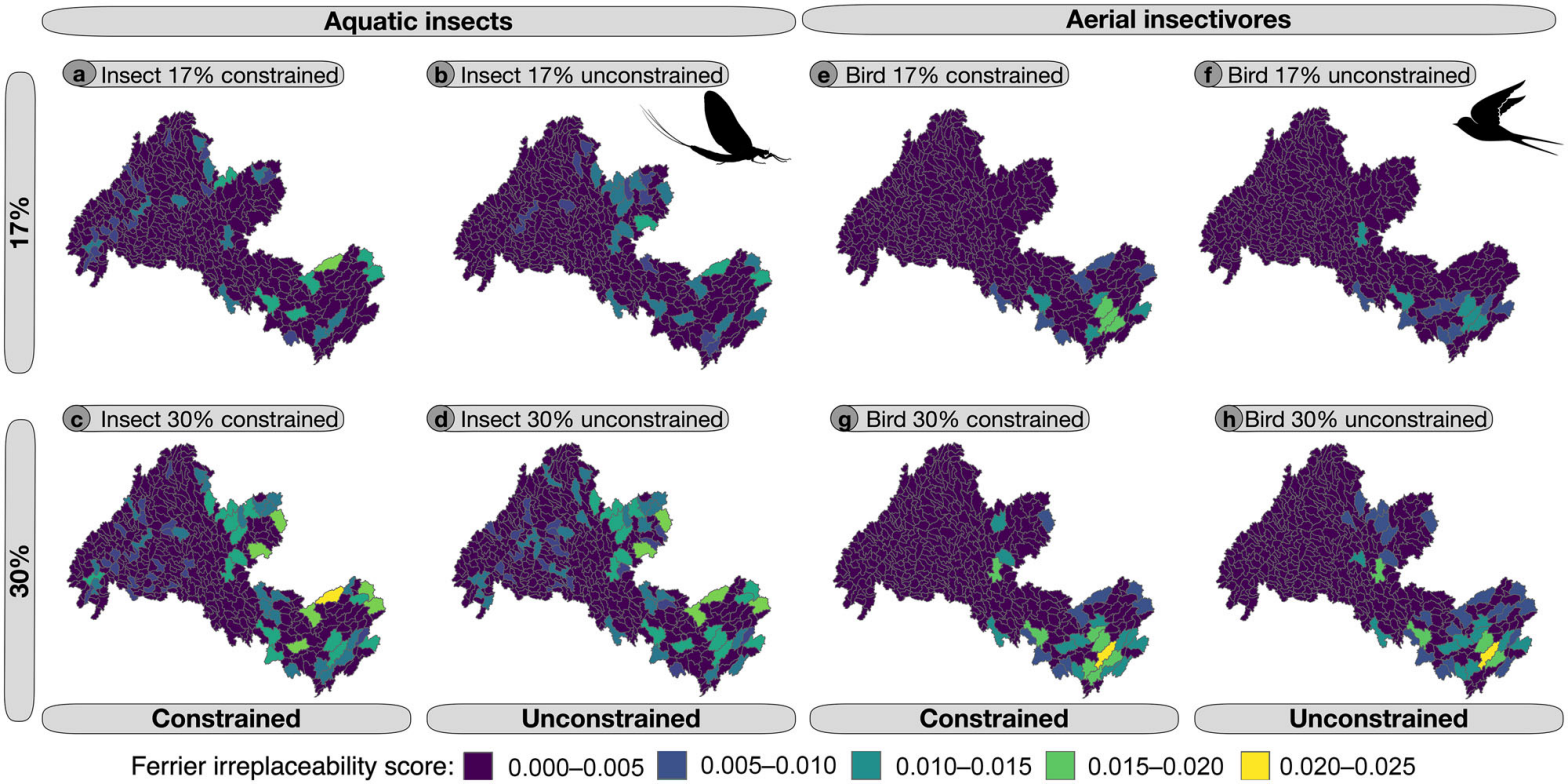
Minimum set prioritization results showing selected sites with irreplaceability scores for (a–d) aquatic insects and (e–h) aerial insectivores for 17% and 30% conservation targets. Constrained panels show solutions that have conserved lands as locked‐in constraints, whereas unconstrained panels are solutions without constraints.

**FIGURE 6:**
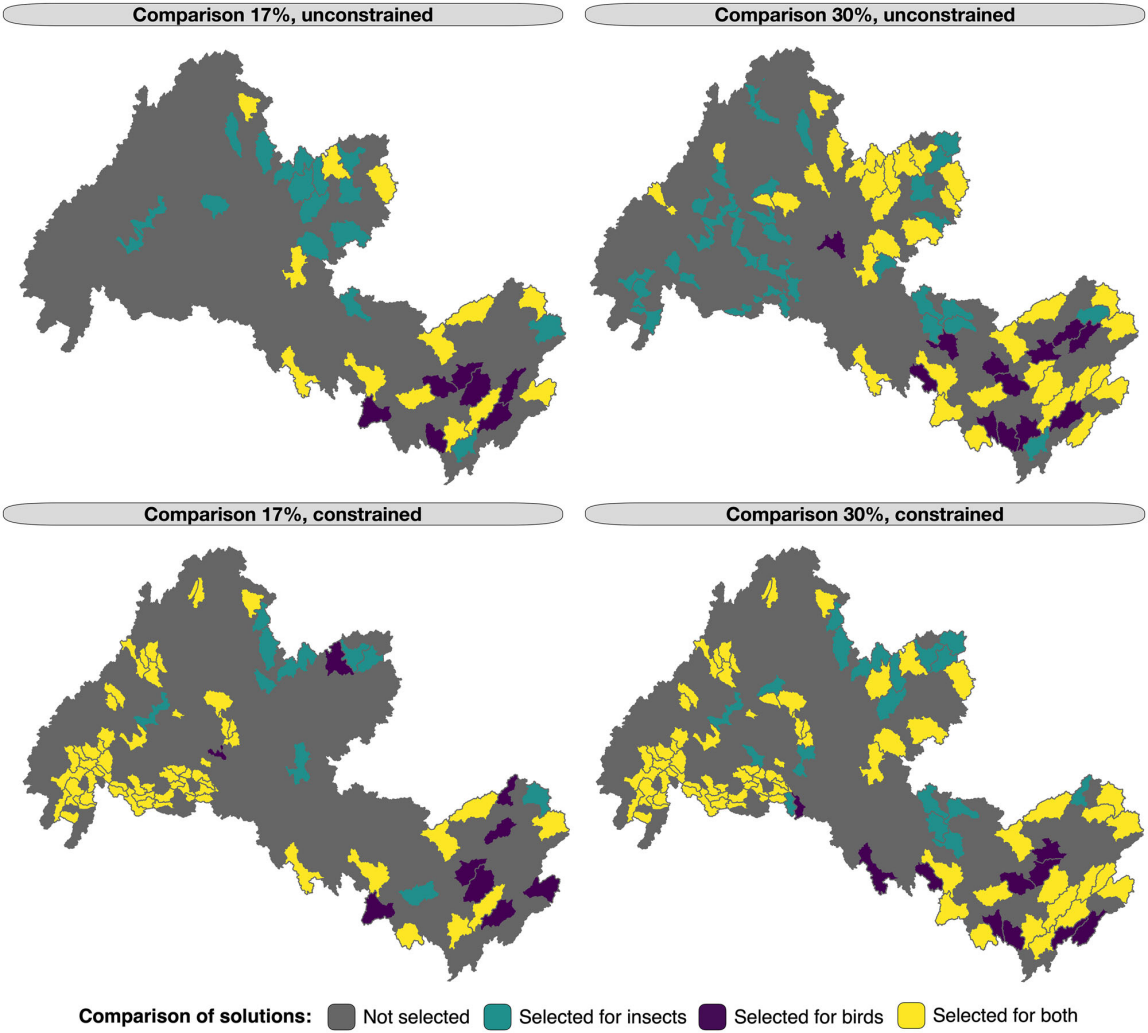
Comparison of aquatic insect and aerial insectivore selected conservation features across the scenarios with either 17% or 30% area conserved and with or without locking in existing protected lands (i.e., constrained or unconstrained).

Although solutions prioritizing aquatic insects showed a uniform selection of hydrologic units across the watershed for both the constrained and unconstrained scenarios, priority areas for birds were more focused on the lower Wolastoq (Figure 5). In general, the major trade‐off between focusing on the prioritization of aquatic insects compared with aerial insectivores was a shift from areas being selected in the headwater regions to a strong focus on habitat along the main stem of the Wolastoq and the lower region of the watershed (Figure 6). Jaccard similarity coefficients between the selected planning units in the scenarios ranged from 0.40 to 0.66 (Appendix S5). Sensitivity (proportion of prioritized units in agreement between both aquatic insects and aerial insectivores) increased as conservation area targets increased and when existing protected areas were locked into the solutions (Appendix S5).

### Conservation value of existing protected lands for freshwater biodiversity and ecosystem function provisioning

When existing protected lands were not included a priori as locked‐in factors in the prioritization (i.e., unconstrained solutions), selected areas for conservation of ecosystem functioning only overlapped with protected lands by a small percentage. The contribution of protected lands to unconstrained solutions averaged at 10.1% for aquatic insects and ranged from 4% for eastern phoebe to 6.47% for tree swallows (Table 1). When conserved lands were included a priori as locked‐in factors, prioritized areas were added mostly in the northwestern (Maine) part of the watershed. For both ecosystem function providers and receivers, hydrologic units with the highest irreplaceability score in the optimization (i.e., most important) were outside of conserved lands and were largely concentrated at the upper portion of the Wolastoq for aquatic insects (e.g., Serpentine River) and at the lower portion for aerial insectivores (e.g., Belleisle Creek), showing that limiting protection to existing protected lands would not be sufficient to protect these taxa under our scenarios. Our data set of existing protected lands did not include land protected under stewardship (e.g., nature preserves), so this metric is likely underestimated.

## DISCUSSION

We conducted a case study for watershed nature‐based restoration, combining aquatic and terrestrial biodiversity with anthropogenic disturbance and conservation status to support habitat provisioning for aerial insectivores. Our results highlight the risk of identifying suboptimal conservation habitats when considering ecosystem function providers or receivers in isolation. Here, priority conservation areas for aquatic insects were primarily located in cold, headwater streams, whereas priority areas for aerial insectivores were located along the Wolastoq main stem, including large tributaries and floodplains. The discrepancy between function providers and receivers was highest at the 17% area targets, with 30% targets showing more similarity in selected watershed units. Although we did have eDNA data for the floodplain of the Wolastoq, much of our insect data represented the biodiversity of lotic taxa, with wetlands not yet covered under biomonitoring programs in the region. Because aerial insectivores forage in open areas (e.g., croplands) and floodplains, it is possible that these areas experience high secondary production via aquatic insects (e.g., Lewis‐Phillips et al., 2020; Raitif et al., 2022) and may be underrepresented in our models and in existing conservation areas. Conservation actions that could benefit both groups could involve the protection of urban and agricultural wetlands to ensure landscape provisioning of high‐quality, nutrient‐rich aquatic insects (Shipley et al., 2022; Twining et al., 2021) and restoration of wetlands to reduce anthropogenic stressors, such as contaminants that may be transferred to foraging aerial insectivores (Alberts et al., 2013; Kraus, 2019).

Previous research in the Wolastoq shows that looking across terrestrial and aquatic realms has value in the long‐term protection of blue–green biodiversity (Camaclang et al., 2021). However, current protected areas in the watershed did not cover the most diverse regions for either aerial insectivores or aquatic insects. Moreover, existing protected areas in the watershed were generally small in area, and many watershed units were not identified as protected because the conservation features did not add up to the 30% target. This suggests that the existing conservation network is suboptimal for aerial insectivores that forage on a wider landscape scale, such as swallows, which seek out ephemeral clusters of insect prey (Garrett et al., 2022; Kusack et al., 2022). Identified conservation lands in the Maine part of the upper watershed were large but not critical for the focal aerial insectivore taxa. These lands could, however, represent areas of high aquatic insect biodiversity, which may yet be underrepresented in this area due to the limited availability of eDNA data.

This discrepancy between biodiversity hotspots and existing conserved areas indicates that ecosystem functioning could be at risk as threats from climate warming and other human pressures expand. The Wolastoq retains a relatively intact and functioning floodplain—rare for large rivers of the Global North (Tockner & Stanford, 2002). However, climate warming is predicted to increase the frequency and magnitude of large floods (McGlynn et al., 2024), potentially disrupting the wetland habitats of lower floodplains, which were identified here as priority habitats for aerial insectivores. Previous shifts in wetland habitats of the lower Wolastoq have been documented and are attributed in part to hydroelectric dam operation and highway construction (Rideout, Compson et al., 2021). In contrast, headwater streams were associated with high aquatic insect richness, yet these streams are at risk of hydrological alteration from expanding forestry practices (Jin et al., 2023) and may exhibit loss of cold‐water habitat under climate change (Linnansaari et al., 2023). This disparity between conservation protection, threats, and biodiversity can inform conservationists about the watershed's vulnerability to future stresses as well as the recovery potential under nature‐based restoration activities (Ahn & Kim, 2019).

Conservation and restoration in localized areas of the watershed, such as small headwater streams, can have positive impacts on the larger watershed and associated metacommunity. Emerging aquatic insects provide a nutrient‐dense food source for aerial insectivore populations in prioritized areas and they can also disperse to other areas of the watershed that may be targets for nature‐based restoration. Aquatic insects can disperse passively (e.g., on the wind) or actively (flying) throughout riparian and terrestrial ecosystems; however, most emerging individuals are deposited within 100 m of a stream, making lateral connections between watershed units less likely than longitudinal connections along the river network (Parkyn & Smith, 2011). Parkyn and Smith (2011) suggest that maintaining or restoring riparian forests along river networks is important to connect more pristine headwater streams with downstream restoration areas as many dispersing aquatic insects will use the river network for dispersal (although some taxa, such as stoneflies, will disperse upstream). By conserving headwater areas and promoting connectivity across habitat patches, the entire watershed can benefit. This contrasts with the field‐of‐dreams approach of traditional restoration (Hilderbrand et al., 2005; Palmer et al., 1997), where practitioners focus on the reconstruction of the physical environment with the assumption that biodiversity will be restored. Rather, by integrating metacommunity theory, longitudinal and lateral habitat connectivity, and ecosystem functioning, areas throughout the watershed can undergo nature‐based restoration at nested spatial scales (e.g., Bullock et al., 2022; Montoya et al., 2012). Our 2 focal groups are less affected by in‐stream connectivity constraints, such as dams, that limit the distribution and dispersal of other aquatic taxa including fish. To include these taxa, our approach could be expanded to include more direct measures of connectivity across the river network (e.g., Finn et al., 2023; Hermoso et al., 2012).

By integrating SDMs of different habitat guilds or taxonomic groups into prioritization schemes, previous studies have quantified the trade‐offs between alternative conservation solutions for multiple threatened species of grassland and forest bird communities (de Zwaan et al., 2022) and priority areas for conservation of species interactions (Heinen et al., 2020). For example, Nogueira et al. (2023) showed that areas important for the interaction between threatened freshwater mussels and their host fish in the Douro River basin (Iberian Peninsula) tend to be underrepresented when data on both taxonomic groups were not simultaneously included in the spatial prioritization. This is consistent with our findings, which showed that species interactions at the aquatic–terrestrial interface tend to be underrepresented in conservation solutions that focus on prioritizing habitats only for ecosystem function providers relative to ecosystem function receivers, and vice versa. One major advantage of spatial prioritization in conservation is the ability to integrate multiple data layers, such as multiple species, taxonomic groups, ecosystem functions, and habitat structure, along with the human footprint (e.g., Morán‐Ordóñez et al., 2022; Ramel et al., 2020; Southee et al., 2021). Using this multilayered information, efficient restoration efforts should focus on providers and receivers of selected ecosystem functions to better balance trade‐offs between small, wadeable streams (where most restoration activity typically focuses) and large complex floodplain habitats that may be identified as priority habitats for conservation of aerial insectivores and other beneficiaries of secondary production.

Our model outputs identified potentially degraded areas of low richness in the watershed that may be candidates for nature‐based restoration. We also identified conservation and high‐biodiversity areas that can provide source habitats for recolonization of adjacent affected areas (van Reese et al., 2023), including areas where restoration would be less feasible (e.g., where urban land costs may be prohibitive). Other researchers have also identified potential rewilding areas based on human footprint, with the assumption that areas with lower anthropogenic stress could be restored more rapidly (e.g., Araújo & Alagador, 2024). Pilot projects or small‐scale restoration efforts can be informed by the results of spatial prioritization analyses to maximize ecological benefits and connectivity between habitats and scaled up to broader landscapes and regions. In the United Kingdom, Brown et al. (2024) explored spatial prioritization under a range of future climatic and socioeconomic conditions until 2080 and identified potential rewilding areas beyond current U.K. government targets. They caution that high‐resolution spatial and ecological modeling (<1 km^2^) would still be required to support decision‐making. Nature‐based restoration through land abandonment or active removal of anthropogenic stressors has been proposed as a viable strategy to meet nature conservation goals in Europe, including 30% area targets (Araújo & Alagador, 2024). Aligning with our suggestions, Araújo and Alagador (2024) recommend that maintaining dispersal pathways and restoring landscape connectivity between planning units are vital to successful nature‐based restoration (also discussed in Pereira et al., 2024).

This process of prioritizing areas for restoration and conservation can help support a thriving landscape, where ecosystem functions are prioritized and protected, while still allowing people and communities to remain active users and stewards of the land. This is a stark contrast to historical, colonial approaches to the establishment of conservation areas, where communities, especially rural and Indigenous, are (or were at risk of being) removed from the landscape (Dotson & Pereira, 2022). Further, these more dated approaches viewed wilderness as not synonymous with human habitation and use (Dotson & Pereira, 2022). The more recent move to working in partnership with stakeholders and rights holders on the implementation of restoration actions is clearly also vital to the long‐term success of watershed healing (Ogar et al., 2020). Local communities are often more invested, with higher stakes in ensuring sustainable management of land and resources (Lanjouw, 2021). Using 3 case studies focused on the Danube River floodplain, Tschikof et al. (2024) integrated ecosystem service multifunctionality with stakeholder participatory engagement to form science‐based decisions related to goals and sites for prioritization of management, conservation, and restoration. Some regional differences in stakeholder perceptions were found; but overall, assessments of this nature need an adaptive and iterative exchange across scales and approaches (i.e., top‐down vs. bottom‐up) to ensure that any decision‐support process can be done collaboratively (Tschikof et al., 2024).

Rewilding and restoration projects must balance the granularity of observation against the availability of data when identifying project goals because there is a general mismatch between data quantity, quality, and density versus availability, particularly when working at larger spatial scales (Schulte to Bühne et al., 2022; Thompson et al., 2018). This problem of data deficiency can be addressed through the creation of proxy data (Eigenbrod et al., 2010). In our study, we used high‐resolution environmental DNA data as a proxy for secondary productivity because direct observational data did not exist at the watershed scale. Data gaps can also be filled through larger scale data collaborations and approaches (e.g., GeoBON) or by pairing sparse observations with high‐resolution geospatial data to support a wider scale of observation. Examples include using imagery collected through satellite remote sensing, terrestrial radar systems, or unmanned aerial vehicles equipped with multispectral sensors (e.g., van Klink et al., 2022). For example, Stepanian et al. (2020) identified aquatic insect population declines by analyzing multiyear emerging insect abundance with radar remote sensing, showcasing how these methods can be used for monitoring ecosystem functioning at large scales. Technological advancements in machine learning and ecoacoustics are now generating large‐scale data sets to support biodiversity monitoring, including for aquatic and terrestrial insects (Linke et al., 2018; van der Lee et al., 2020; van Klink et al., 2022). Expanding the use of advanced monitoring technologies, such as environmental DNA (eDNA), can rapidly provide high‐resolution data on biodiversity and ecosystem functions at large spatial scales, allowing for more precise and informed decision‐making. Most importantly, the view of information needs to be broadened, and different ways of knowing need to be adopted to support a holistic concept of ecosystem functioning. By respectfully bridging knowledge systems, decision‐making can be strengthened with a stronger evidence base at extended temporal and spatial scales (Alexander et al., 2021). Working closely with local communities, stakeholders, and Indigenous groups to ensure that conservation strategies are socially acceptable and beneficial aids the integration of socioecological systems into conservation planning. Comanagement approaches that involve these groups can enhance the success and sustainability of restoration projects (e.g., Diver, 2016; Freitas et al., 2019; Ma et al., 2023).

In reference to prioritizing habitat for 30×30, Eckert et al. (2023) proposed that a national target (as opposed to region or provincially based targets) can maximize terrestrial biodiversity and that the Maritime provinces, including New Brunswick, where the Wolastoq is primarily located, should have a much greater representation in this conservation goal. In the WWF Wildlife Protection Assessment, the province of New Brunswick was rated very poor, with only 1% of physical habitats adequately protected (WWF‐Canada, 2019). Despite its ecological importance, the report identified the Wolastoq as being almost entirely unprotected or inadequately protected. Additionally, given the transboundary nature of many ecosystems, including the Wolastoq watershed, there is a need for cross‐jurisdictional collaboration in conservation planning. Policies and management practices should be harmonized across regions to ensure the protection and restoration of shared ecosystems. While working across aquatic–terrestrial boundaries at a watershed scale, we have proposed solutions that can fuel discussions about how ecosystem functioning and nature‐based restoration can help to work toward Canada's 30×30 goal.

Despite the limitations discussed above, our approach to modeling habitat provisioning addresses broad concerns regarding data adequacy for exploring large‐scale area‐based conservation targets (e.g., Jetz et al., 2021), which can be limited by poor spatial coverage and inconsistent observation of multispecies targets. By combining citizen science and environmental DNA observations and geospatial data, we demonstrated the potential to map potential hotspots of critical biodiversity and ecosystem function. This has a 2‐fold benefit: it supports status assessment of current conserved lands and it indicates areas in a region that have the capacity to provide critical ecosystem services to surrounding areas to support future nature‐based restoration efforts (e.g., Rideout, Wegscheider et al., 2021). Although we focused on the Wolastoq, our approach could be easily applied to other bioregions and habitat types. Moreover, we strongly support the value of unifying conservation and restoration efforts across freshwater and terrestrial habitats, and watersheds can provide a natural unit for the focus of conservation and restoration efforts at a regional scale. In the future, conservation planning must include the protection of cross‐ecosystem fluxes of energy and materials, which, until recently, have received minimal consideration in target setting.

## Supporting information



Supplementary 1: DNA metabarcoding workflow.
Supplementary 2: Evaluation of multi‐taxon distribution model performance.
Supplementary 3: Evaluation of spatial prioritization.
Supplementary 4: Aquatic insect richness and habitat.
Supplementary 5: Comparison of prioritization between conservation solutions for aquatic insects and aerial insectivores.
