# Supplementary material for: Modeling nature‐based restoration potential across aquatic–terrestrial boundaries
Source: Conserv Biol. 2025 Apr 28;39(5):e70046. doi: 10.1111/cobi.70046 (PMC12451488; doi:10.1111/cobi.70046)
Supplement: Supplementary file 1 — Supplementary 1: DNA metabarcoding workflow. Supplementary 2: Evaluation of multi‐taxon distribution model performance. Supplementary 3: Evaluation of spatial prioritization. Supplementary 4: Aquatic insect richness and habitat. Supplementary 5: Comparison of prioritization between conservation solutions for aquatic insects and aerial insectivores. [file COBI-39-e70046-s001.docx]

**Supplementary 1: DNA metabarcoding workflow**

**Table S1.1** Details of the combined insect observational data set in terms of numbers of samples collected and amplicons used for taxonomic assignment. See text for further details.

|  | **River samples** | **Floodplain samples** |
| --- | --- | --- |
| Number of samples | 81 | 60 |
| Amplicons sequenced | F230, FWH2, BR5 | F230, BR5 |

*DFO detailed molecular protocols*

1. **Fieldwork**

We employed some minor modifications of traditional protocols for operators to minimize DNA contamination. These include wearing nitrile gloves to handle DNA samples (particularly if excess vegetation or rocks need to be removed from the sample), and treating sampling equipment with diluted household bleach (1 in 10 dilution resulting in about 0.5% sodium hypochlorite) between surveys and during sample processing to avoid cross-contamination. After collection, samples destined for sequencing should be stored in ≥ 95% ethanol and then as a precaution, kept as cold as possible in the field, during storage and in shipping to prevent the DNA from degrading, with long term storage in a spark-proof freezer at -20 °C, or, if possible -80 °C. Our experience suggests that as long as about 60% or more of the container volume is ethanol, samples can be packed with ice or stored at ambient temperature in the field, and stored for weeks at room temperature without consequential deterioration

**2.**    **Lab**

**2.1 Field Sample Homogenization**

*Protocol*:

1.    Prior to processing, the workstation and all equipment are thoroughly cleaned to remove traces of DNA, typically using bleach solution (1 in 10 dilution of household bleach), and then rinsed thoroughly with water.

2.    Small rocks approximately 0.5 cm in diameter and larger, and excess vegetation can be rinsed with ethanol to collect invertebrates before removing them from the sample.

3.    If samples are large or contain a high proportion of ethanol, the mixture is drained through a sterile mesh of the same mesh diameter as the field net to minimize volume to be blended.

4.    Samples are blended for 1 to 5 minutes at ~100-120 revolutions per second using a Waring® blender until well homogenized.

5.    Two to three 30 mL subsamples (in 50mL tubes) are then retained from this mixture for extraction and future use.

6.    The remaining mixture of ethanol and the blended sample is disposed of in accordance with guidelines for hazardous waste and all glassware is cleaned and treated with bleach solution as above before use with another sample.

**2.2 DNA Extraction**

*Protocol*:

After step 3 below, we employed the DNeasy PowerSoil Kit (Qiagen, Catalog No. 12888-100) following the manufacturer’s protocol.

Ethanol evaporation:

1.    Centrifuge samples in Falcon tubes at maximum speed for 5 minutes. If no Falcon-tube compatible centrifuge is available you can allow the material to settle to the bottom overnight.

2.    Remove the supernatant by decanting and/or pipetting.

3.    Stir the pellet, and let dry in a water bath (under the fume hood) at 60°C for 2 to 3 hours until slightly cracked and dry. The samples are stirred midway through.
 Note: dry samples can be frozen overnight at -20°C until you are ready to continue.

DNA extraction:

4.    Approximately 250 mg of homogenized field sample is transferred to the PowerBead Tubes.

5.    Add 60 uL of Solution C1 (heat to 60°C if precipitate is visible)

6.    Vortex tubes for 10 minutes at max speed (using a horizontal adaptor).

7.    Centrifuge at 10,000 x g for 30 seconds at room temperature.

8.    Transfer supernatant (400-500uL) to clean 2 mL collection tube.

9.    Add 250 uL of Solution C2 and vortex for 5 seconds. Incubate at 4°C for 5 minutes.

10.  Centrifuge at 10,000 x g for 30 seconds at room temperature.

11.  Avoiding the pellet, transfer supernatant (up to 600 uL) to clean 2 mL collection tube.

12.  Add 200 uL of Solution C3 and vortex for 5 seconds. Incubate at 4°C for 5 minutes.

13.  Centrifuge at 10,000 x g for 1 minute at room temperature.

14.  Avoiding the pellet, transfer the supernatant (up to 750 uL) to a clean 2 mL collection tube.

15.  Shake to mix Solution C4. Add 1,200 uL of Solution C4 to the supernatant and vortex for 5 seconds.

16.  Load 650 uL onto a Spin Filter and centrifuge at 10,000 x g for 1 minute at room temperature.

17.  Discard flow through.

18.  Add an additional 650 uL onto a Spin Filter and centrifuge at 10,000 x g for 1 minute at room temperature.

19.  Add the remainder of the sample (approximately 650 uL) onto a Spin Filter and centrifuge at 10,000 x g for 1 minute at room temperature.

20.  Discard flow through.

21.  Add 500 uL of Solution C5, centrifuge at 10,000 x g for 30 seconds at room temperature.

22.  Discard flow through.

23.  Centrifuge at 10,000 x g for 1 minute at room temperature.

24.  Transfer Spin Filter into a new collection tube.

25.  Add 30 uL of Solution C6 to the center of the filter membrane.

26.  Wait 1 minute.

27.  Centrifuge at 10,000 x g for 1 minute at room temperature.

28.  Add another 30 uL of Solution C6 to the center of the filter membrane.

29.  Wait 1 minute.

30.  Centrifuge at 10,000 x g for 1 minute at room temperature.

31.  Discard Spin Filter.

32.  Store DNA at -20C.

**2.3 Preliminary Progress Check: DNA Quantification and Normalization**

*Protocol*:

DNA is quantified using the Qubit 2.0 Fluorometer (Life Technologies, MODEL NO.Q32866) using 1 ul of DNA post-extraction as spectrophotometric methods can have low accuracy in the presence of inhibitors. Samples are normalized to 10 ng/uL, or left as is if the concentration is below 10 ng/uL.

**2.4 Primer selection**

*Protocol*:

We selected 3 primer pairs to amplify 3 separate COI loci in freshwater invertebrates for the GRDI Eco-biomics project (Edge et al 2020).

The primers used in the current workflow include the F230 primers from Gibson *et al.* (2015), however, the reverse primer was modified to use “N” rather than inosine because following the PCR product for the former was better on agarose gel. We also used fwh2 and BF2-BR2 primers (Elbrecht & Leese 2017; Vamos et al., 2017), because they each identified a significant unique proportion of the community that was complementary to the other, and to F230 (i.e. a number of taxa were only identified by one primer). A two-step PCR approach was used to prepare the Illumina-competent amplicon libraries.

**2.5 PCR Step 1**

*Protocol*:

For PCR step 1, each sample is amplified for F230, fwh2 and BF2 in separate reactions (giving approximate fragment sizes of 347 bp, 321 bp and 528 bp respectively after PCR step 1). A ratio of 4:1 of tailed and non-tailed primers is used for the first PCR as we determined that the inclusion of non-tailed primers increased the amplification efficiency considerably (data not shown). For the tailed primers, a staggered approach was used to generate sequence diversity in the first rounds of sequencing, with a different proportion of each staggered oligonucleotide depending on the amplicon. Please note that the ratios used of the different staggered primers were optimized to ensure high base diversity in the first 12 bp of read 1. You may want to change the ratio of the different staggered primers if fewer loci are pooled together on one run or if you use different primers entirely.

For the first round of PCR, Amplitaq Gold 360 Mastermix (Applied Biosystems, Catalog No. 4398881) was used due to its robustness in the presence of inhibitors (data not shown) for all three amplicons following the protocol below and the primers in Table S1.2 in a total volume of 25 µl. A total of 5 (fwh2) or 6 (BF-BR2 and F230) oligonucleotides are used in separate reactions for the three loci. The tailed primers were pooled together based on proportions shown in Table S1.2, and 1.2 µl of this mix was added to each reaction.

PCR reaction setup:

For each individual reaction combine:

5.5 µL of H_2_O

                    12.5 µL of Amplitaq Gold 360 mastermix

                    1.2 µL of Forward ‘’tailed’’ primers mix (20 µm stock concentration)

Table X in bold for ratios used)

1.2 µL of Reverse ‘’tailed’’ primers (20 µm stock concentration)

0.3 µL of Forward ‘’non-tailed’’ primers (20 µm stock concentration)

0.3 µL of Reverse ‘’non-tailed’’ primers (20 µm stock concentration)

1uL of BSA 1%

3 µL of DNA

F230 and fwh2 were amplified following these PCR conditions: initial incubation at 95°C for 10 min; 30 cycles of denaturation at 95°C for 30 sec; annealing at 46°C for 30 sec; extension at 60°C for 60 sec; and, a final extension at 72°C for 7 min.  BF2 was amplified using the same conditions except the annealing was at 48°C instead of 46°C.

**Table S1.2:** Forward and reverse primers currently used by the Ecobiomics project, including tailed sequences in bold.

| **Amplicon/ Primer name** | **Forward (5’→3’)** | **Volume of 20 µM** |
| --- | --- | --- |
| BF2 |  |  |
| BF2 (non-tailed) | GCHCCHGAYATRGCHTTYCC | 0.3 µl |
| BR2 (non-tailed) | TCDGGRTGNCCRAARAAYCA | 0.3 µl |
| BF2_no_Stagger_tailed (33%) | TCGTCGGCAGCGTCAGATGTGTATAAGAGACAGGCHCCHGAYATRGCHTTYCC | 0.4 µl |
| BF2_Stagger_Y_tailed (33%) | TCGTCGGCAGCGTCAGATGTGTATAAGAGACAGYGCHCCHGAYATRGCHTTYCC | 0.4 µl |
| BF2_Stagger_TC_tailed (33%) | TCGTCGGCAGCGTCAGATGTGTATAAGAGACAGTCGCHCCHGAYATRGCHTTYCC | 0.4 µl |
| BR2_tailed | GTCTCGTGGGCTCGGAGATGTGTATAAGAGACAGTCDGGRTGNCCRAARAAYCA | 1.2 µl |
| Fwh2 |  |  |
| fwhF2 (non-tailed) | GGDACWGGWTGAACWGTWTAYCCHCC | 0.3 µl |
| fwhR2n (non-tailed) | GTRATWGCHCCDGCTARWACWGG | 0.3 µl |
| fwhF2_no_Stagger_tailed (66%) | TCGTCGGCAGCGTCAGATGTGTATAAGAGACAGGGDACWGGWTGAACWGTWTAYCCHCC | 0.8 µl |
| fwhF2_neg_Stagger_tailed (33%) | TCGTCGGCAGCGTCAGATGTGTATAAGAGACAGGDACWGGWTGAACWGTWTAYCCHCC | 0.4 µl |
| fwhR2n_tailed | GTCTCGTGGGCTCGGAGATGTGTATAAGAGACAGGTRATWGCHCCDGCTARWACWGG | 1.2 µl |
| F230 |  |  |
| LCO1490-Folmar-F (non-tailed) | GGTCAACAAATCATAAAGATATTGG | 0.3 µl |
| F230R_modN (non-tailed) | CTTATRTTRTTTATNCGNGGRAANGC | 0.3 µl |
| LCO_1490_Folmar_no_Stagger_ tailed (33%) | TCGTCGGCAGCGTCAGATGTGTATAAGAGACAGGGTCAACAAATCATAAAGATATTGG | 0.4 µl |
| LCO_1490_Folmar _stagger_C_tailed (33%) | TCGTCGGCAGCGTCAGATGTGTATAAGAGACAGCGGTCAACAAATCATAAAGATATTGG | 0.4 µl |

*PCR Step 1 Clean-up*: After the PCR step 1, a clean-up was performed using High Prep PCR magnetic beads (MAGBIO, Catalog. No. AC-60050) following the manufacturer’s protocol.  A ratio of 1.5X of HighPrep PCR reagent was added to the PCR reaction and DNA was resuspended in 60 µL of Tris-HCl 10 mM pH 8.0.

**2.6 PCR Step 2:**

For the second round of PCR, the Q5 Hot start High-fidelity DNA polymerase (New England BioLabs, Catalog No.M0493S) was used for all three amplicons following the protocol below and the primers listed in Table S1.2. Unique combinations of different forward and reverse 8 bp index sequences (denoted by X’s) are used for each sample, which enables bioinformatic de-multiplexing of individual samples (Table S.1.3). Unused tag combinations can be created (tag-jumping) from used tags if samples are pooled, potentially resulting in the mis-assignment of sequences to the correct samples. However, as samples in our protocol are not pooled until after the final round of PCR, the opportunity for tag-jumping is almost eliminated.

**Table S1.3.** Forward and reverse 8 bp index sequences (denoted by X’s) used for each sample (see 2.6 above).

| **Primer name** | | **Forward (5’→3’)** |
| --- | --- | --- |
| i5-forward index | AATGATACGGCGACCACCGAGATCTACACXXXXXXXX**TCGTCGGCAGCGTC** | |
| i7-reverse index | CAAGCAGAAGACGGCATACGAGATXXXXXXXX**GTCTCGTGGGCTCGG** | |

Each reaction was performed using 12.0uL of H_2_O, 0.25 µL of Q5 Hot start High-fidelity DNA polymerase, 5 µL of 5X Q5 reaction buffer, 0.5 µL of 10 nM dNTPs (deoxynucleotides solution), 0.625 µL of Forward Index primer (20 µm stock concentration), 0.625 µL of Reverse Index primer (20 µm stock concentration), 1 uL of 1% BSA and 5 uL of cleaned DNA from PCR step 1. F230, BF2-BR2 and fwh2 were amplified following these PCR conditions: initial incubation at 98°C for 30 sec; 10 cycles of denaturation at 98°C for 15 sec; annealing at 66°C for 20 sec; extension at 72°C for 30 sec; and, a final extension at 72°C for 2 min. After the PCR step 2, fragments are visualized on 1.5% agarose gel by loading 5 uL of each sample. There should only be one band for each amplicon of approximately 416 bp for F230, 390 bp for fwh2 and 597 bp for BF2.

**2.7 Secondary Progress Check: DNA Quantification**

*Protocol*:

Quantify some or all samples using the Qubit fluorometer to estimate the amount of DNA in each sample. The normalization kit manual recommends loading 5-25 uL of PCR products with a total of at least 250 ng amplicon/well.

**3.8 Quality Control: Normalization**

*Protocol*: Samples were normalized using the SequalPrep Normalization Plate (ThermoFisher Scientific, Catalog. No. A1051001) following the manufacturer’s protocol.

**3.9 Sample Pooling**

*Protocol*:

Pipette 20 µl of each eluate from the normalization plate into a single 5 ml tube. This can be done using a multichannel pipette and sterile buffer reservoir or empty tube strip for larger sample numbers.

**3.10 Bead clean-up to concentrate DNA**

Pooled samples are cleaned using a ratio of 1.2x of High Prep PCR magnetic beads following the manufacturer’s protocol as mentioned above. DNA is resuspended in 40 uL of Tris-HCl 10 mM, pH 8.0.

**3.11 Size selection of pools using Pippen Prep**

In order to minimize off-target amplification products, we used a Pippin Prep DNA size selection system (Sage Sciences, Model No. PIP0001) and the 2% gel cassette (D-Mark Biosciences, Catolog. No. CDF2010) targeting fragments ± 125 bp of expected final fragment size.

**3.12 Determination of pool quality and quantity**

After size selection, each pool was quantified with the Qubit HS DNA kit and concentrated if needed using a Vacufuge plus (Eppendorf, Model No. 5305).

Samples were placed on an Experion Electrophoresis Station (BioRad, Model No.) to confirm elimination of off-target amplicons and expected fragment size. The concentration of each pool was also determined via qPCR.

**Supplementary 2: Evaluation of multi-taxon distribution model performance**

We derived a set of metrics from a contingency matrix containing true positives , true negatives , false positives and false negatives. Specifically we estimated the True skill statistic (TSS), KAPPA and Accuracy metrics. In addition, we estimated the threshold-independent area under the receiver operative characteristic curve (AUC).


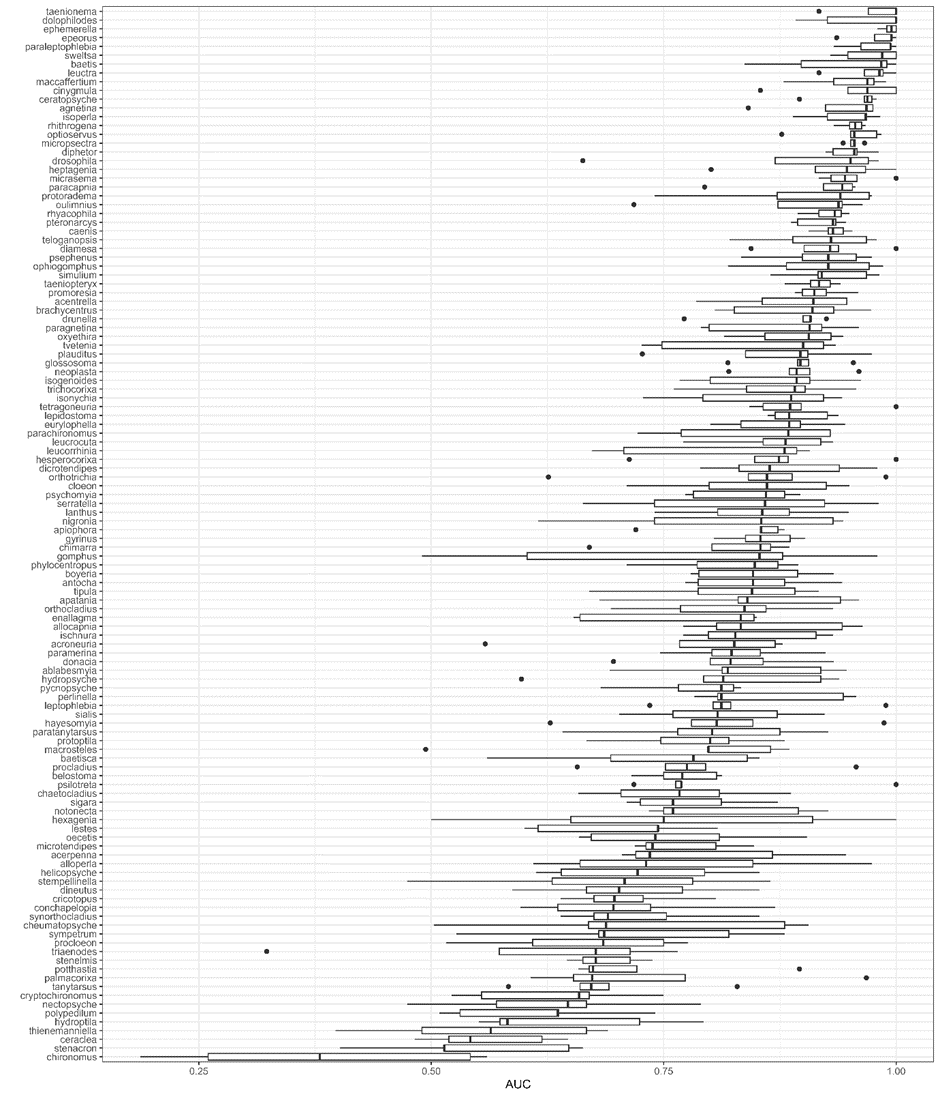
**Figure S2.1** Genus-specific summary for model performance of aquatic insects using Area under the Receiver Operating Characteristic Curve (AUC) as an evaluation metric.


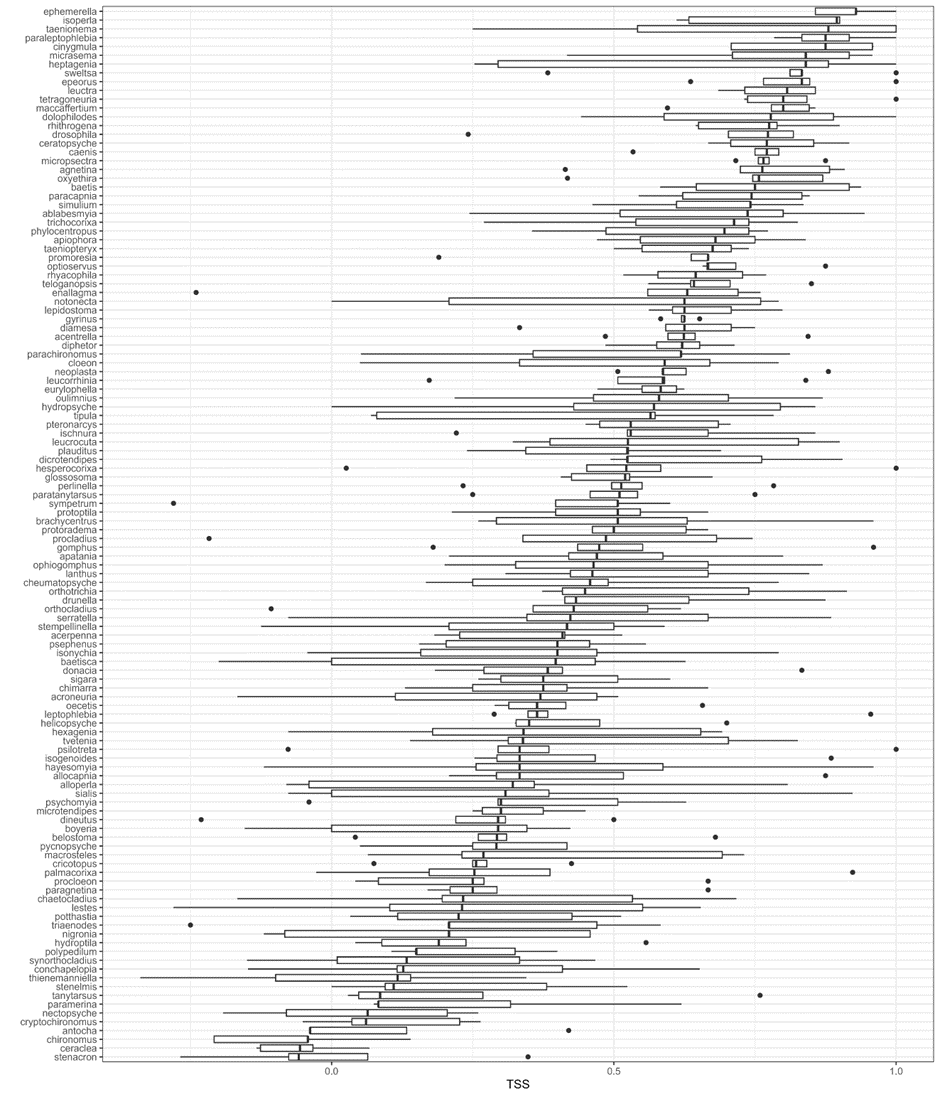
**Figure S2.2** Summary of species distribution models for 120 aquatic insect genera using the True Skill Statistic (TSS) as an evaluation metric for model performance


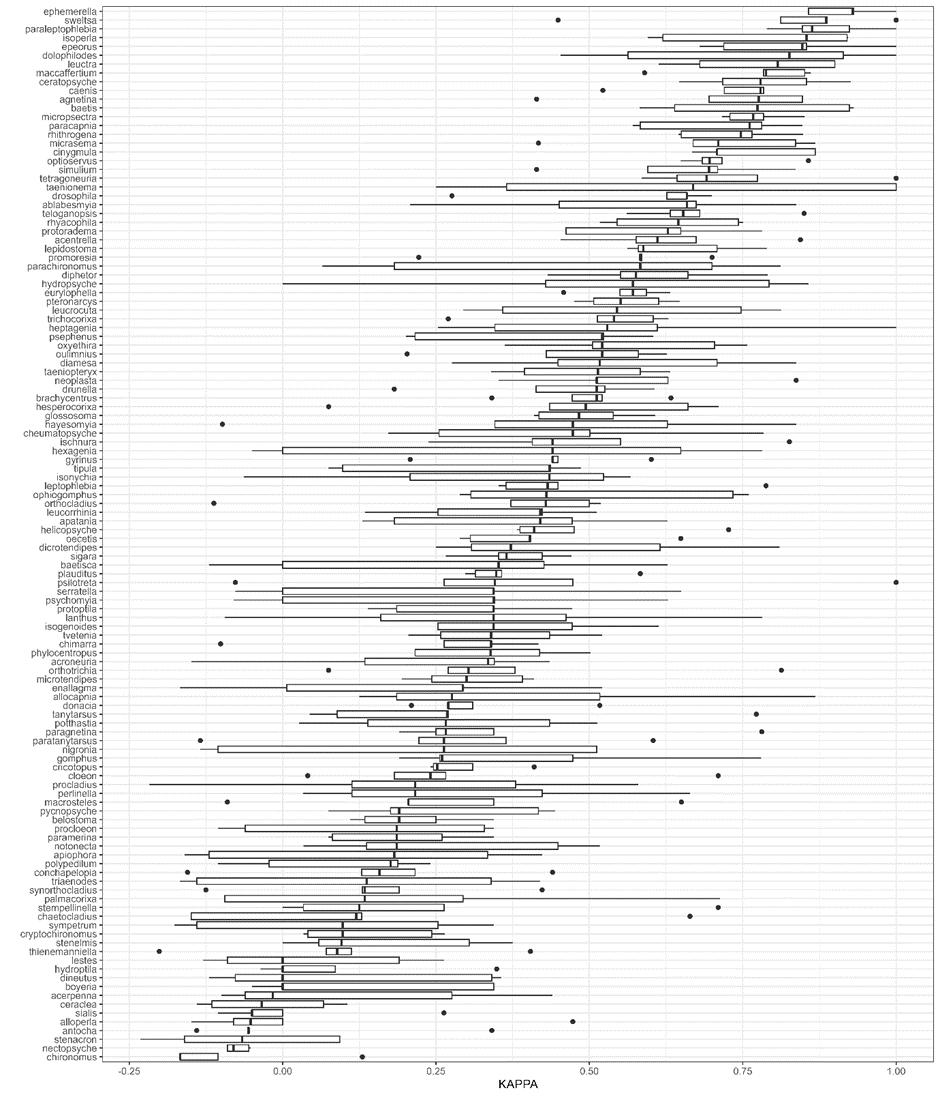
**Figure S2.3** Summary of species distribution models for 120 aquatic insect genera using Cohen`s Kappa statistic as an evaluation metric for model performance


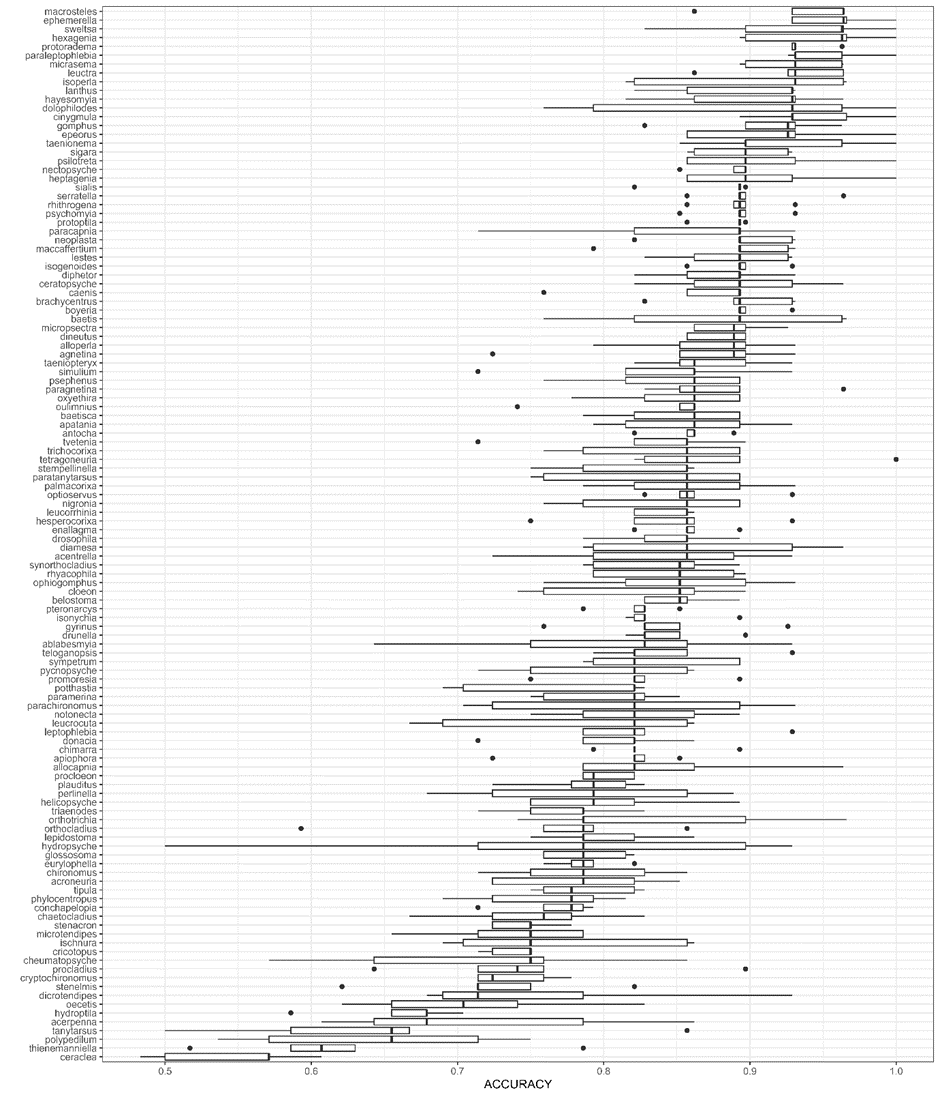
**Figure S2.4** Summary of species distribution models for 120 aquatic insect genera using the Accuracy statistic as an evaluation metric for model performance


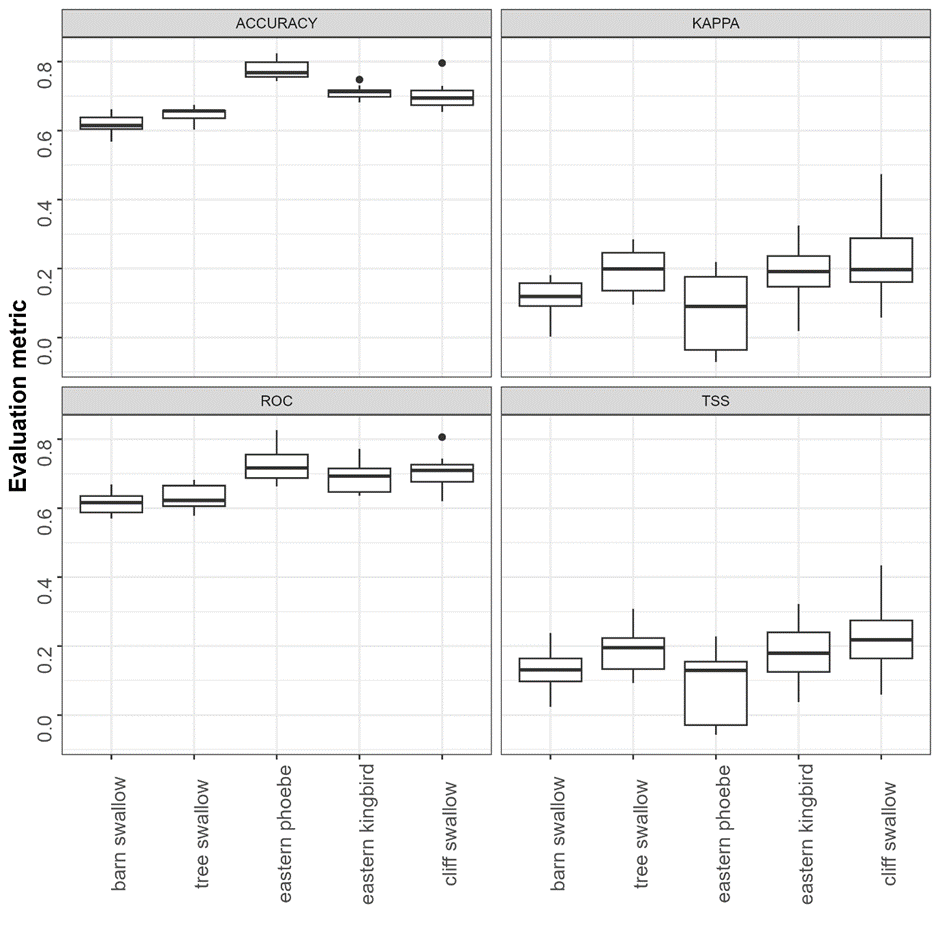
**Figure S2.5.** Species-specific summary for model performance of aerial insectivores using Accuracy, the Area under the receiver operating characteristic curve (AUC), Cohen’s Kappa, and True Skill Statistic (TSS) as an evaluation metric.

**Supplementary 3: Evaluation of spatial prioritization**

We tested whether the conversion of probability to binary values resulted in the selection of different priority areas, and derived summary statistics from a confusion matrix for a quantitative comparison. Overall the prioritizations showed similar solutions for both types of conservation features, with large overlap in both and planning units were consistently selected or not selected (see Sensitivity and Specificity metrics in Table S2.1). Therefore, we conclude that selected priority areas for aquatic insects represent truly important habitats and are not an aggregate of multiple planning units with low habitat suitability.


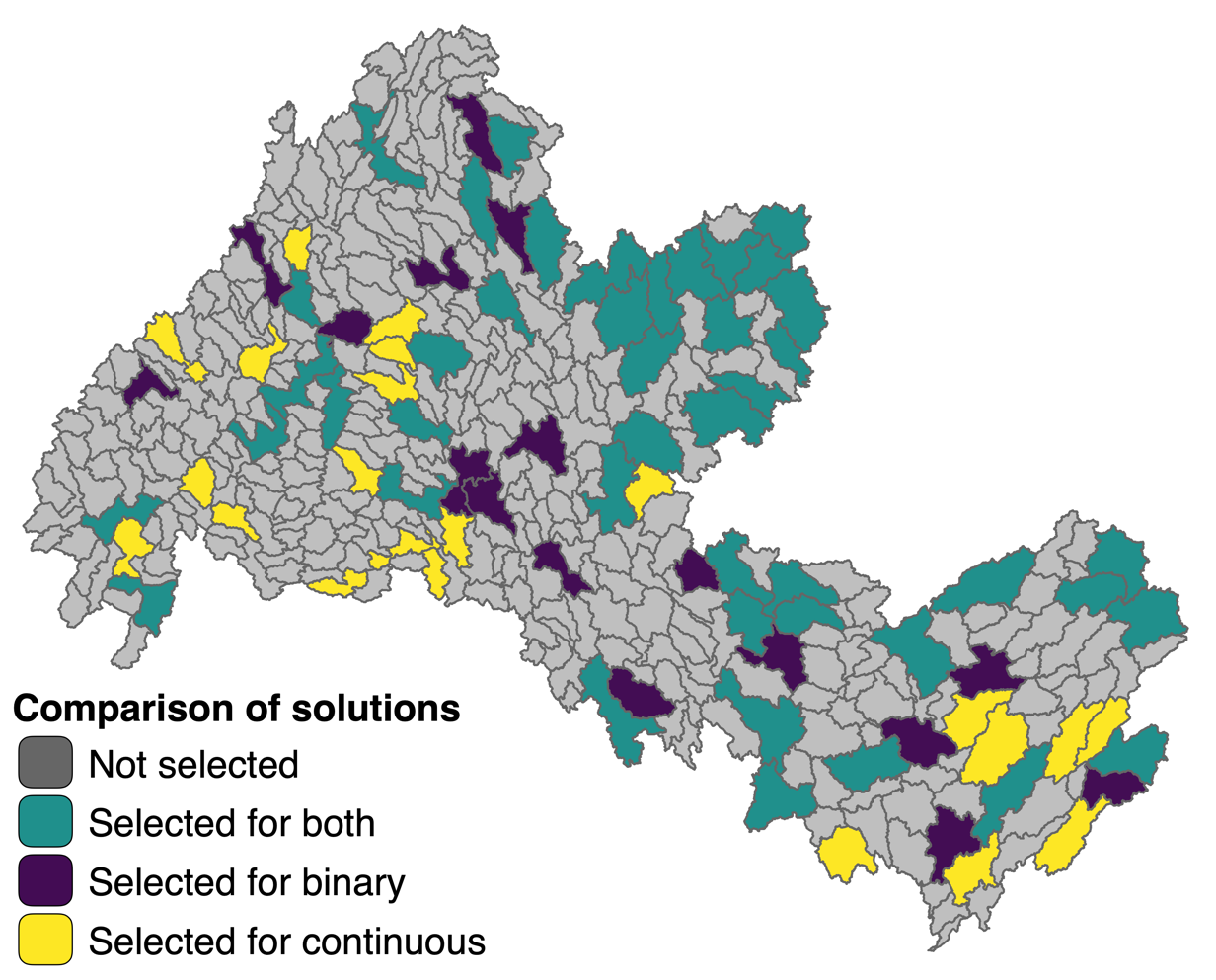


**Figure S3.1** Comparison of prioritizations based on continuous vs binary conservation features for aquatic insects, with the spatial target set at 30%.

**
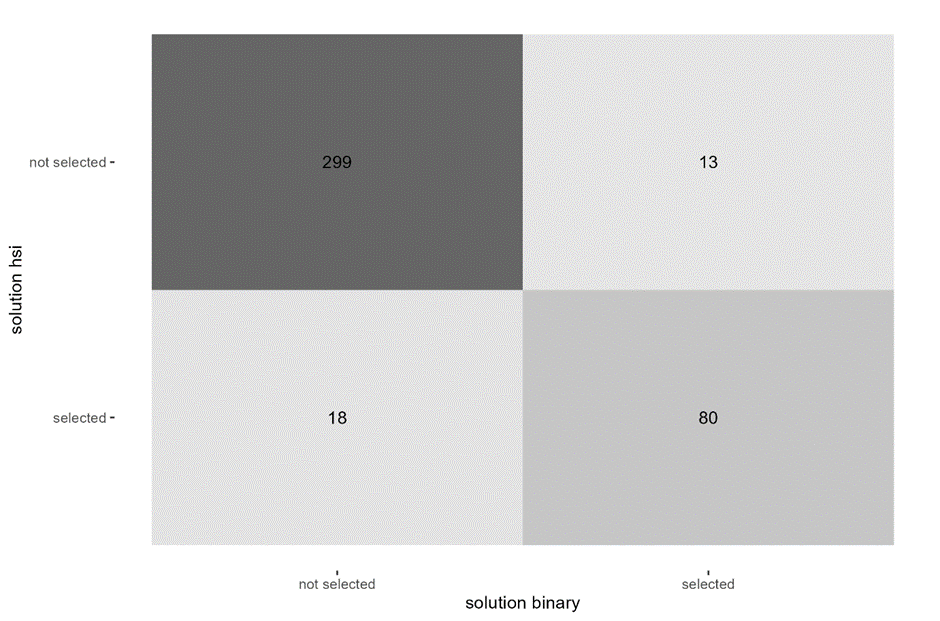
Figure S3.2** Confusion matrix for the selection of planning units (n=410) as priority areas in a scenario with binary and continuous predictions of 94 aquatic insect genera as conservation features, 30% representation targets and protected areas as locked-in constraints.


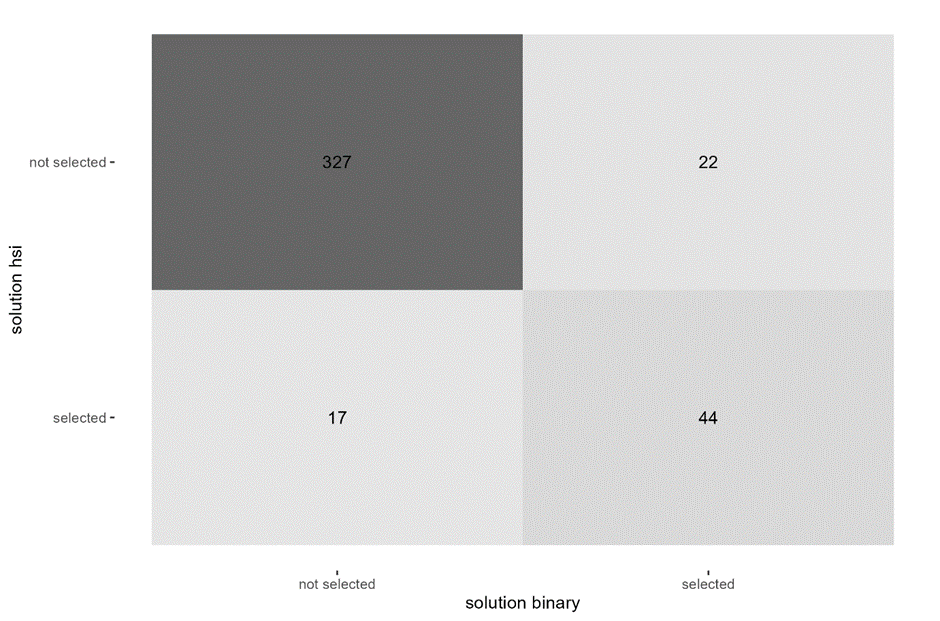
**Figure S3.3** Confusion matrix for the selection of planning units (n=410) as priority areas in a scenario with binary and continuous predictions of 94 aquatic insect genera as conservation features, 30% representation targets and without locked-in constraints.
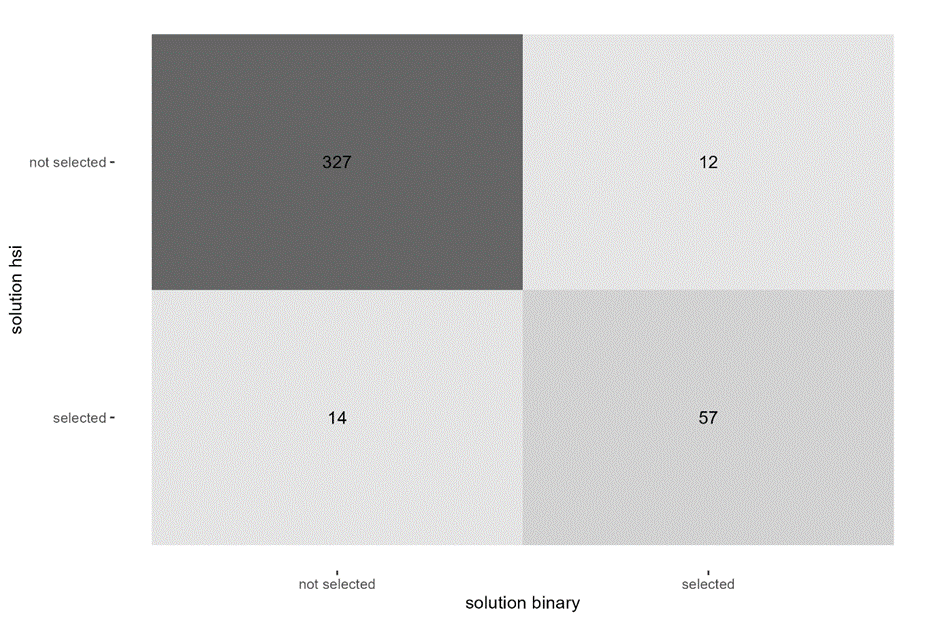
**Figure S3.4** Confusion matrix for the selection of planning units (n=410) as priority areas in a scenario with binary and continuous predictions of 94 aquatic insect genera as conservation features, 17% representation targets and protected areas as locked-in constraints.


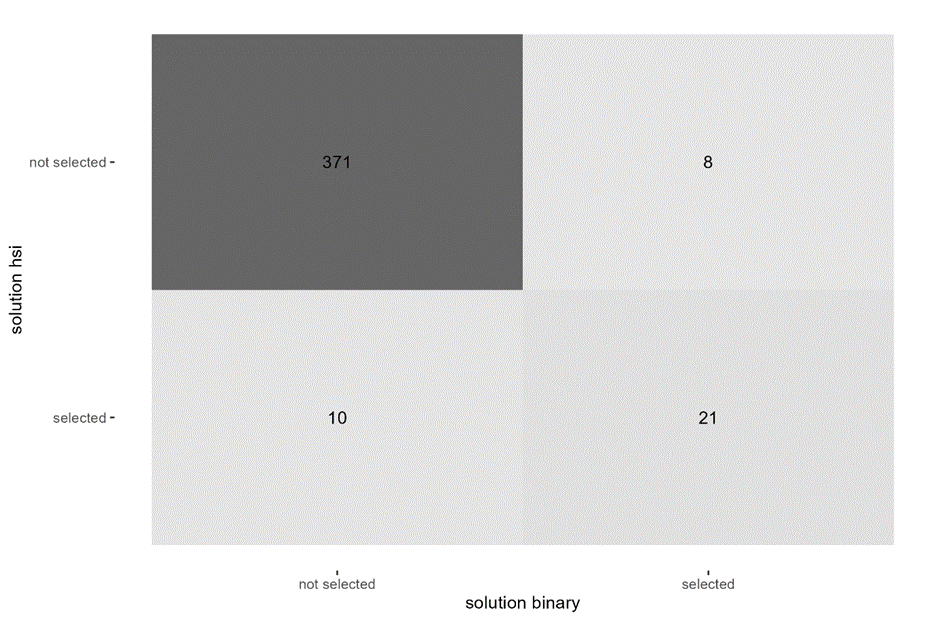
**Figure S3.5** Confusion matrix for the selection of planning units (n=410) as priority areas in a scenario with binary and continuous predictions of 94 aquatic insect genera as conservation features, 17% representation targets and no locked-in constraints.

**Table S3.1** Evaluation metrics derived from a confusion matrix comparing prioritizations using binary (0/1) versus continuous predictions of probability of occurrence as conservation features. Sensitivity and Specificity represents the proportion of priority areas that were consistently selected or not selected in both scenarios, respectively. Kappa is a statistical measure ranging from 0-1, used to assess the agreement between the two prioritizations, correcting for chance. The Jaccard similarity coefficient evaluates the percentage number equal to the intersection between two solutions, divided by the size of the union of these solutions. A Kappa and a Jaccard value of 1 would represent perfect agreement, while a value of 0 would represent inverse selection of planning units between both prioritizations.

| **Scenario** | **Sensitivity [%]** | **Specificity [%]** | **Kappa** | **Jaccard Index** |
| --- | --- | --- | --- | --- |
| 17%, unconstrained | 68 | 98 | 0.68 | 0.58 |
| 17%, constrained | 80 | 96 | 0.78 | 0.49 |
| 30%, unconstrained | 72 | 91 | 0.63 | 0.62 |
| 30%, constrained | 81 | 95 | 0.79 | 0.66 |

**Supplementary 4: Aquatic insect richness and habitat**

**Figure 4.1** Predicted focal genus richness of (a) aquatic insects and (b) their habitat associations predicting genus richness in the Wolastoq watershed, Canada. (points, average genus richness for each subcatchment relative to individual environmental predictors).

**
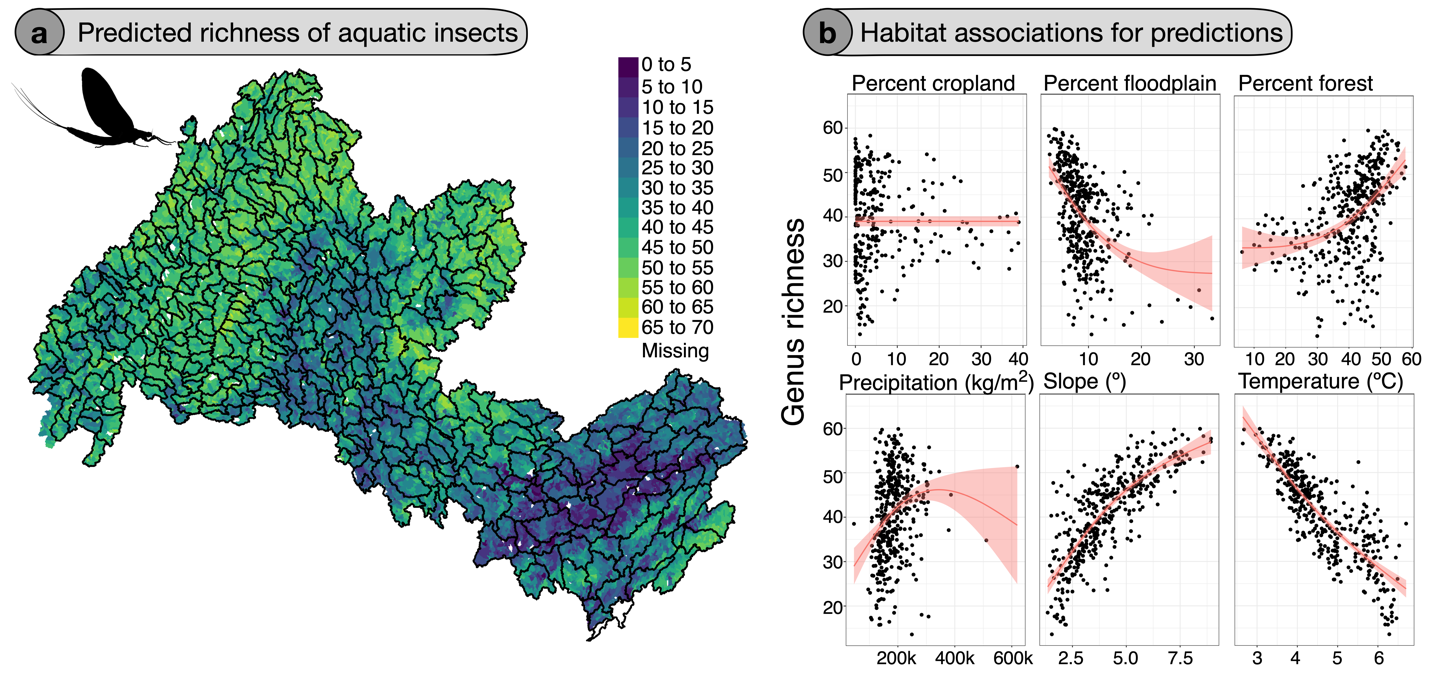
**

**Supplementary 5: Comparison of prioritization between conservation solutions for aquatic insects and aerial insectivores.**

| Scenario | Sensitivity (%)^[[1]](#footnote-1)^ | Specificity [%]^[[2]](#footnote-2)^ | Kappa^[[3]](#footnote-3)^ | Jaccard index^[[4]](#footnote-4)^ |
| --- | --- | --- | --- | --- |
| 17%, unconstrained | 65 | 95 | 0.50 | 0.40 |
| 17%, constrained | 87 | 97 | 0.83 | 0.61 |
| 30%, unconstrained | 74 | 91 | 0.55 | 0.53 |
| 30%, constrained | 88 | 94 | 0.80 | 0.66 |

1. Proportion of priority areas consistently selected in both scenarios [↑](#footnote-ref-1)
2. Proportion of priority areas consistently not selected in both scenarios [↑](#footnote-ref-2)
3. Range 0-1; assesses agreement between the two prioritizations, correcting for chance, where 1 is perfect agreement [↑](#footnote-ref-3)
4. Range 0-1; evaluates the percentage number equal to the intersection between two solutions divided by the size of the union of these solutions, where 1 is perfect agreement [↑](#footnote-ref-4)
